# Superior capsule reconstruction, partial cuff repair, graft interposition, arthroscopic debridement or balloon spacers for large and massive irreparable rotator cuff tears: a systematic review and meta-analysis

**DOI:** 10.1186/s13018-022-03411-y

**Published:** 2022-12-19

**Authors:** Andrew Davies, Prashant Singh, Peter Reilly, Sanjeeve Sabharwal, Amar Malhas

**Affiliations:** 1 Cutrale Perioperative and Aging Group, Department of Bioengineering, Imperial College London, 86 Wood Lane, London, W120BZ UK; 2 Department of Orthopaedics, Imperial College Healthcare NHS Trust, London, UK; 3 Department of Orthopaedics, Royal Berkshire NHS Foundation Trust, Reading, UK

**Keywords:** Rotator cuff, Rotator cuff tears, Injuries, tendon, Shoulder

## Abstract

**Background:**

Multiple non-arthroplasty surgical techniques are described for the management of large and massive irreparable rotator cuff tears. There is currently no consensus on the best management strategy. Our aim was to compare clinical outcomes following arthroscopic debridement, arthroscopic partial cuff repair, superior capsule reconstruction, balloon spacers or graft interposition for the management of large and massive irreparable rotator cuff tears.

**Methods:**

A comprehensive search was performed of the following databases: Medline, Embase, CINAHL and Cochrane Database of Systematic Reviews. Data were extracted from relevant studies published since January 2000 according to the pre-specified inclusion criteria. The primary outcome was the post-operative improvement in shoulder scores. Meta-analysis of the primary outcome was performed. Secondary outcomes included retear rates and complications.

**Results:**

Eighty-two studies were included reporting the outcomes of 2790 shoulders. Fifty-one studies were included in the meta-analysis of the primary outcome. The definition of an irreparable tear varied. All procedures resulted in improved shoulder scores at early follow-up. Shoulder scores declined after 2 years following balloon spacers, arthroscopic debridement and partial cuff repair. High retear rates were seen with partial cuff repairs (45%), graft interposition (21%) and superior capsule reconstruction (21%).

**Conclusions:**

Large initial improvements in shoulder scores were demonstrated for all techniques despite high retear rates for reconstructive procedures. Shoulder scores may decline at mid- to long-term follow-up.

## Background

Rotator cuff tears are a common cause of shoulder pain and can lead to significant functional impairment [1]. A primary consideration for large and massive tears is whether the tendon can be repaired. A tendon may be considered irreparable when it cannot be mobilised to the anatomical footprint despite mobilisation techniques such as interval slide and marginal convergence [2]. Poor tendon architecture with a high degree of fatty infiltration will also reduce the likelihood of a successful repair.

Several surgical techniques have evolved for the management of large and massive irreparable tears in adult patients without significant arthritic disease. Partial cuff repair (PCR) is an option where complete repairs are not possible [3]. Reconstruction of infraspinatus and teres minor is prioritised to re-establish the transverse force couple. Further techniques have evolved including subacromial balloon spacers, superior capsule reconstruction (SCR) and the use of autografts, allografts, xenografts or synthetic grafts to bridge residual cuff defects [2].

SCR aims to prevent superior migration of the humeral head and restore normal shoulder biomechanics; it involves passing a graft from the superior tubercle of the glenoid to the greater tuberosity [4, 5]. Similarly, balloon spacers are designed to prevent superior migration by deploying biodegradable saline-filled spacers underneath the acromion. Several large medical device companies have invested in the technology, and there has been a rapid expansion in data from case series [6].

Several tendon transfer techniques have been described to restore shoulder function according to tear configuration [7]. Posterosuperior tears may be managed with latissimus dorsi or trapezius tendon transfers [7, 8]. These have been shown to be effective in select groups; they are most commonly used in younger, high-demand patients and require specialised rehabilitation programmes [7, 8]. As a consequence, the patient populations managed with tendon transfers will likely be different compared to the other procedures in this review and tendon transfers will not be considered further. Reverse total shoulder replacement can lead to significant improvement in function and pain in patients with concurrent arthritis; however, concern about the complication and revision rates has limited its application in those without advanced arthritis, particularly in younger patients [9].

Despite the significant impact on patients’ quality of life, socio-economic considerations and protracted rehabilitation periods, a consensus on the optimal strategy has not been reached. Studies have demonstrated positive patient-reported outcome measures (PROM), functional scores and reduced pain for arthroscopic debridement, arthroscopic PCR, SCR, subacromial balloon spacers and graft interposition.

### Aim

The aim of this review is to answer the following research question: In adult patients with a large or massive irreparable tear of the rotator cuff does arthroscopic debridement, arthroscopic PCR, SCR, subacromial balloon spacer or graft interposition lead to the greatest improvement in shoulder scores and the lowest risk of complications? Randomised trials and observational studies were considered. Our hypothesis is that SCR, balloon spacer and graft interposition do not result in superior shoulder scores compared to arthroscopic PCR.

## Methods

The review protocol was registered on the International Prospective Register of Systematic Reviews (PROSPERO) database. The review was conducted according to the Preferred Reporting Items for Systematic Reviews and Meta-Analyses (PRISMA) Statement [10].

### Search strategy

The following databases were used: Medline, EMBASE, CINAHL and the Cochrane database of systematic reviews, from inception until May 2021. Trial registries were searched to highlight ongoing work. A scoping literature review was performed prior to protocol submission to optimise the search terms and inclusion criteria. The search terms are related to the population (rotator cuff tear, supraspinatus, infraspinatus, subscapularis, teres minor tears, AND massive, large, irreparable) and the interventions (superior capsule reconstruction, partial cuff repair, balloon spacer, graft bridging, debridement). Reference lists were scrutinised for further work. The full search terms are available in the Appendix.

### Eligibility criteria

The review included adult patients with large or massive rotator cuff tears that were considered irreparable. The reparability of the cuff was determined by the authors of each study based on pre-operative imaging and intra-operative assessment. Across all studies, this was defined as a tear where the tendon could not be brought back to the tendon insertion without excessive tension on the tendon.

A minimum tear size was required; large or massive tears were included. Large and massive tears were defined as 3 cm or greater in any dimension or involving two or more tendons. The studies must report on pre-operative and post-operative shoulder scores for any of the included procedures: arthroscopic debridement, arthroscopic PCR, SCR, subacromial balloon spacers and graft interposition of incomplete repairs. Graft interposition was defined as cases where a graft was used to bridge a remaining tendon defect. It did not include cases where grafts were used in addition to a complete repair. In SCR, the graft is anchored into the superior glenoid and the greater tuberosity. Articles published no earlier than 2000 were considered. All types of graft material for SCR and graft interposition were accepted. Randomised trials and observational studies with multiple or single intervention groups of ten or more patients were included. Where trials compared one or more techniques that matched our inclusion criteria against alternative procedures, data from the relevant treatment arm were extracted.

Articles were excluded if they reported on tears associated with additional trauma including fractures or dislocations and patients with documented cuff tear arthropathy, SLAP tears, or advanced glenohumeral osteoarthritis. Small and medium tears (< 3 cm) were excluded alongside procedures that involved additional bone marrow infiltration, complete repair or augmentation of a complete repair. Animal or in vitro work, editorials, conference abstracts, case reports, case series of less than 10 patients and letters were also excluded. We included studies where patients may have undergone a previous failed rotator cuff repair. Finally, we required a minimum of 12 months of follow-up. We excluded studies with less than 12 months follow-up, where earlier outcome data could not be separated.

### Outcomes

The primary outcomes were PROMs and functional scores including, but not limited to the Constant-Murley score, American Shoulder and Elbow Score (ASES), Oxford Shoulder Score (OSS), University of California in Los Angeles (UCLA) Shoulder Score, the Disabilities of Arm, Shoulder and Hand score (DASH), Subjective Shoulder Value (SSV), Simple Shoulder Test (SST) or the Japanese Orthopaedic Association (JOA) shoulder score. Secondary outcomes include retear rates and complications.

### Study selection

Two authors (AD and PS) independently assessed trials for eligibility. Search results were organised using Covidence® systematic review management software. Duplicates were removed, and articles were selected according to the inclusion criteria. Conflicts were discussed between reviewers; a third reviewer (SS) was available as required to arbitrate.

### Data extraction

A standardised form guided data extraction in four areas: study characteristics, pre-operative information, details of the interventions and post-operative outcomes (Table 1). Data were extracted by AD and PS, and the results were checked independently by both authors. In order to be included in the quantitative synthesis, studies must have reported PROMs as mean (± standard deviation). To avoid error, data were not extracted from graphs. To pool PROMs across studies, outcome duration was grouped into 1 year, 2 years, 3 years and 5 or more years. The durations of follow-up were frequently presented as a mean and range. To standardise the analysis, all patients within a group must have achieved the minimum follow-up duration in order to be included.

**Table 1:** Data extraction. OSS—Oxford Shoulder Score, JOA—Japanese Orthopaedic Association score

Study characteristics	Study design, institutions, no. of relevant procedures, no. of centres, date of publication, number of patients, funding source, sampling methods
Pre-operative information	Patient demographics and co-morbidities, pre-operative functional scores, quality of life assessments, diagnostic criteria, pre-operative imaging
Intervention details	The surgical approaches, types of grafts and implants, patient position, method of insertion
Post-operative outcomes	PROMs and functional scores, complication rates, post-operative management

### Quality and bias assessment

A risk-of-bias assessment was performed in all studies by two authors (AD and PS) with consensus reached by discussion where necessary (Appendix 4). The Methodological Index for Non-randomized Studies (MINORS) instrument was chosen following publication of the protocol because all except 1 study were observational and the majority were case series [11]. This instrument includes an eight-item assessment of non-comparative studies. The instrument can be extended to 12 items in order to include comparative studies.

### Data analysis

Pre-operative and post-operative PROMs and functional scores were extracted. The difference between the pre-operative and post-operative scores was calculated at 1, 2, 3 and 5 years post-procedure. Direct comparison between procedures was not made given the quality of the available literature, outcomes for each procedure were pooled independently. To account for the different scores, the standardised mean difference (SMD) was calculated and weighted according to sample size. A random effects model was selected due to the variability in study design.

To facilitate interpretation of the SMD, this was re-expressed in the units of the Constant-Murley score. This was the score most commonly used across included studies. Transformation was performed according to the Cochrane handbook [12]. We used the pooled standard deviation of all studies included in the quantitative synthesis that reported the Constant-Murley score. Analysis was performed using Review Manager 5.3. Mean differences were calculated for the continuous data. Heterogeneity was assessed using the I^2^ Chi^2^ and Tau^2^ statistics.

## Results

**Fig. 1:**
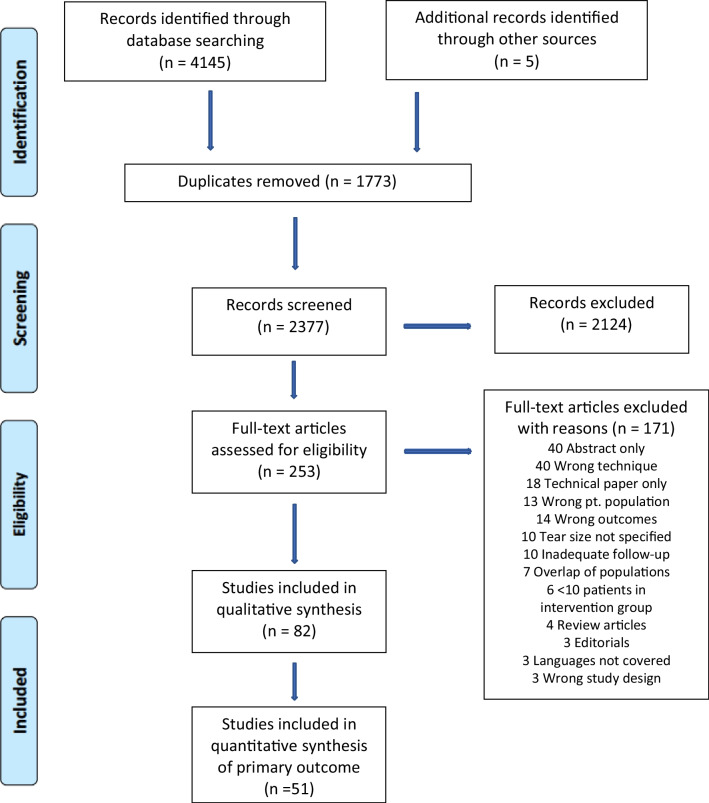
PRISMA study flow diagram

The search strategy revealed 4145 results; 5 further potential studies were identified through screening of references. After removal of duplicates, 2377 articles were available for screening (Fig. 1). Screening of titles and abstracts excluded 2124 articles. One hundred and seventy-one articles were excluded on full-text review leaving 82 studies for qualitative synthesis. Fifty-one studies were included in the quantitative synthesis of the primary outcome.

### Study characteristics

Of the 82 eligible studies, 60 were case series, 21 were cohort studies and 1 was a randomised controlled trial (Tables 2, 3, 4, 5 and 6). The surgical management of 2790 shoulders was reported. The majority of studies were performed in a single centre; 5 involved patients from two or more institutions. Sufficient data for quantitative synthesis were provided in 51 studies. This includes 37 case series and 14 comparative studies (13 cohort studies and 1 randomised trial), where one or more of the treatment groups were included. In four cohort studies and in the randomised trial, one or more groups did not meet the inclusion criteria and data were extracted for the relevant management arm.

**Table 2:** Partial cuff repair—included studies

Author and date	Design	Mean age	No. of shoulders at f/u—PROMs	Follow-up duration (months)	Study results pooled	Functional/PROMs outcomes measured
Baverel [13]	Cohort–PCR vs LDTT	68.5	26	16–29	Yes	Constant, ASES, SSV, SST
Berth [14]	Cohort–PCR vs debridement	63.4	21	21–28	Yes	Constant, QuickDASH
Besnard [15]	Cohort–PCR vs complete	59.3	20	24–120	Yes	Constant
Cavalier [16]	Cohort–PCR vs debridement	67	61	12	No	Constant, ASES, SSV
Chen [17]	Case series	60.3	37	25–59	Yes	ASES
Cuff [18]	Case series	65.2	28	60–90	Yes	ASES, SST
Di Benedetto [19]	Case series	67	31	79	Yes	Constant
Farazdaghi [20]	Cohort–PCR vs complete	63.7	14	95	No	ASES, Penn
Franceschi [21]	Cohort–PCR vs debridement	62	34	60–108	Yes	UCLA
Galasso [22]	Case series	62.7	95	24–148	Yes	Constant, SST
Greiner [23]	Cohort–PCR vs SCR	62.3	20	47–79	No	Constant, DASH
Heuberer [24]	Cohort–PCR vs debridement	67	22	23–70	No	Constant, QuickDASH
Holtby [25]	Cohort–PCR vs complete	67	73	24	No	Constant, ASES
Jeong [26]	Cohort–PCR vs complete	61.9	33	60–120	Yes	UCLA, ASES, SSV
Kim [27]	Case series	62.3	27	36–52	Yes	Constant, SST
Kim [28]	Cohort–PCR vs complete	61.5	19	24	Yes	UCLA, ASES, SST
Lee [29]	Case series	61.2	42	31–78	Yes	UCLA, constant
Malahias [30]	Cohort–PCR vs PCR and balloon	69.7	16	12	Yes	Constant, ASES
Mori [31]	Cohort–PCR vs graft interposition	65.7	24	35.6	No	UCLA, Constant, ASES
Moser [32]	Cohort–PCR vs complete vs debridement (< 10 patients)	62.5	11	24	No	SPADI
Pandey [33]	Cohort–PCR vs graft interposition	58	13	24–60	Yes	Constant, OSS
Park [34]	Cohort–PCR vs augmentation	63.8	37	24–51	Yes	Constant, ASES, KSS
Paribelli [35]	Cohort–PCR vs LDTT	64.9	20	12–60	Yes	UCLA
Porcellini [36]	Case series	63	67	60	Yes	Constant, SST
Shon [37]	Case series	65.9	31	40 ± 14.9	Yes	ASES, SST
Wellmann [38]	Case series	65	38	12	No	Constant

Partial cuff repair—included studies. PCR—partial cuff repair, LDTT—latissimus dorsi tendon transfer, complete–complete cuff repair. Constant—Constant-Murley Score, QuickDASH—Disabilities of the Arm, Shoulder and Hand Score, ASES—American Shoulder and Elbow Score, SSV—Subjective Shoulder Value, SST—Simple Shoulder Test, SPADI—Shoulder Pain and Disability Index, UCLA—The University of California at Los Angeles Shoulder Score, KSS—Korean Shoulder Score, OSS—Oxford Shoulder Score, JOA—Japanese Orthopaedic Association score. For comparative studies, the number of shoulders for the relevant treatment arm is reported

**Table 3:** Graft interposition—included studies. OSS—Oxford Shoulder Score, JOA—Japanese Orthopaedic Association score

Author and date	Design	Mean age	No. of shoulders at f/u—PROMs	Follow-up duration (months)	Study results pooled	Graft type used	Functional/PROMs outcomes measured
Audenaert [39]	Case series	67	39	24–86	No	Mersilene mesh	Constant
Badhe [40]	Case series	66	10	36–60	Yes	Porcine dermal xenograft	Constant
Bond [41]	Case series	54.4	16	12–38	Yes	Human dermal allograft	UCLA, Constant
Dukan [42]	Case series	60.5	23	29.3	Yes	Porcine dermal xenograft	Constant
Gupta [43]	Case series	63	24	29–40	No	Human dermal allograft	Constant
Gupta [44]	Case series	60	27	24–40	No	Porcine dermal xenograft	Constant
Kim [45]	Case series	56	24	24–48	No	Human dermal allograft	UCLA, ASES, SST
Kokkalis [46]	Case series	58	21	33–72	Yes	Human dermal allograft	ASES
Modi [47]	Case series	62.6	61	12–72	No	Human dermal allograft	OSS
Mori [31]	Cohort–graft interposition vs PCR	65.7	24	35.6	No	Fascia lata autograft	UCLA, Constant, ASES
Nada 2010[48]	Case series	66.5	21	36	No	Polyester fibre mesh	Constant
Pandey [33]	Cohort–graft interposition vs PCR	58	13	24–60	Yes	Human dermal allograft	Constant, OSS
Petrie [49]	Case series	67.2	31	24	No	Polyester fibre mesh	OSS
Rhee [50]	Case series	61	31	24–67	No	Biceps autograft	UCLA, Constant
Rhee [51]	Case series	66.9	24	12	Yes	Biceps autograft	ASES, QuickDASH
Sano [52]	Case series	64	14	12–51	Yes	Biceps autograft	JOA
Dimitrios [53]	Case series	64.9	68	31–77	Yes	Fascia lata autograft	Constant
Wong [54]	Case series	53.6	45	24–68	No	Human dermal allograft	UCLA

**Table 4:** Superior capsule reconstruction—included studies

Author and date	Design	Mean age	No. of shoulders at f/u—PROMs	Follow-up duration (months)	Study results pooled	Graft type used	Functional/PROMs outcomes measured
Alarcon [55]	Case series	61	31	24–51	No	Fascia lata autograft	Constant
Barth [56]	Cohort—SCR vs complete vs augmentation	60	24	24–29	Yes	LHB autograft	Constant, ASES, SST
Burkhart [57]	Case series	64	41	24–50	Yes	Human dermal allograft	ASES, SSV
Denard [58]	Case series	62	59	12–29	Yes	Human dermal allograft	ASES, SSV,
Ferrando [59]	Case series	65	52	34	Yes	Porcine dermal xenograft	ASES, SSV
Greiner [23]	Cohort–SCR vs PCR	62.3	20	47–79	No	Porcine dermal xenograft	Constant, DASH
Kim [60]	Case series	58	45	24–48	No	LHB autograft	Constant, ASES
Kocaoglu [61]	Cohort–SCR vs SCR and PCR	63.7	26	18–40	Yes	Fascia lata autograft	Constant, QuickDASH
LaBelle [62]	Case series	62.5	28	24–41	Yes	Dermal allograft	SST, ASES
Lacheta [63]	Case series	56	21	24–36	No	Human dermal allograft	ASES, QuickDASH
Lee and Min [64]	Case series	60.9	36	24.8	No	Fascia lata autograft and dermal allograft	Constant, ASES
Lim [65]	Case series	65.3	31	12–24	Yes	Fascia lata autograft	Constant, ASES
Mihata [5]	Case series	65.1	24	24–51	Yes	Fascia lata autograft	ASES, JOA
Okamura [66]	Cohort–SCR 1 layer graft vs 3 layers	76	35	24–69	No	Teflon graft	ASES
Ohta [67]	Case series	75.3	35	12–62	Yes	Fascia lata autograft	UCLA, JOA
Ozturk [68]	RCT–SCR vs LDTT	62.8	20	31	Yes	Fascia lata autograft	Constant, ASES
Pashuck Pashuck 2020	Case series	58.9	14	23–25	Yes	Human dermal allograft	ASES
Pennington [70]	Case series	59.4	88	16–28	Yes	Human dermal allograft	ASES
Polacek [71]	Case series	60	20	12	Yes	Porcine dermal xenograft	SPADI
Polacek [72]	Case series	61.3	24	12	Yes	Fascia lata autograft	SPADI
Takayama [73]	Cohort–SCR mini open vs arthroscopic	69.7	46	24–66	Yes	Fascia lata autograft	ASES

**Table 5:** Arthroscopic debridement—included studies

Author and date	Design	Mean age	No. of shoulders at f/u—PROMS	Follow-up duration [months]	Study results pooled	Functional/PROMs outcomes measured
Berth [14]	Cohort–Debridement vs PCR	63.4	21	21–28	Yes	Constant, QuickDASH
Blanke [74]	Case series	63.4	59	24–36	No	Constant
Boileau [75]	Case series	68	72	24–76	Yes	Constant
Cavalier [16]	Cohort–Debridement vs PCR	67	26	12	No	Constant, ASES, SSV
Franceschi [21]	Cohort–Debridement vs PCR	62	34	60–108	Yes	UCLA
Heuberer [24]	Cohort–Debridement vs PCR	67	23	23–70	No	Constant, QuickDASH
Klinger [76]	Case series	69	33	24–46	No	Constant
Lee [77]	Case series	62.4	32	24–63	No	Constant, UCLA
Mirzaee [78]	Case series	65	12	12–24	Yes	UCLA
Park [79]	Case series	64	16	84–126	Yes	UCLA, constant
Scheibel [80]	Case series	69	22	20–58	No	Constant
Vad [81]	Case series	61.3	32	24–84	Yes	Shoulder rating questionnaire
Veado [82]	Case series	72	12	12–60	Yes	UCLA
Verhelst [83]	Case series	69.9	32	21–52	Yes	Constant

**Table 6:** Balloon spacer—included studies

Author and Date	Design	Mean age	No. of shoulders at f/u—PROMs	Follow-up duration (months)	Study results pooled	Functional/PROMs outcomes measured
Basat [84]	Case series	64.3	12	38	Yes	Constant
Bakti [85]	Case series	67	26	12–60	No	OSS
Deranlot [86]	Case series	69.8	39	12–36	Yes	Constant
Familiari [87]	Case series	63	51	24–56	Yes	Constant
Gervasi [88]	Case series	74.6	15	12–14	No	Constant, ASES
Gervasi [89]	Case series	73	40	24	Yes	Constant, ASES
Iban [90]	Case series	69 [median]	10	24	No	Constant, SST, QuickDASH
Lorente [91]	Case series	69.4	15	12	No	Constant
Maman [92]	Case series	Unknown	42	12–40	No	Constant
Senekovic [93]	Case series	70.5	18	18–36	Yes	Constant

Twenty-six of the included studies reported shoulder scores following partial cuff repair, and 21 studies reported scores following SCR. Eighteen articles recorded results for graft interposition and outcomes for arthroscopic debridement were reported in 15 studies. However, in one cohort study comparing PCR and arthroscopic debridement, the series in the debridement arm was not included due to there being fewer than 10 patients in this treatment group. Ten studies reported shoulder scores for balloon spacers. The criteria used to define large and massive tears were documented in 58 studies. In the remaining articles, the authors described the tears as “large” or “massive” without further details. Magnetic resonance imaging (MRI) was the most favoured imaging modality; 74 papers reported the use of pre-operative MRI or magnetic resonance arthrogram in diagnosis and classification. The remaining either used ultrasound imaging or an intra-operative assessment. The age range of participants across all studies was 33–90. The Hamada classification of rotator cuff arthropathy was the most frequently used. Several studies were excluded due to lack of reporting of tear size, the majority of these were for balloon spacers.

The terminology used to describe graft interposition varied between studies and included “bridging”, “interposition” and “augmentation”. Careful review of the description of the technique was necessary to ensure the grafts were used to fill the remaining defect after the best possible repair. The most popular interposition graft was human dermal allograft (7 studies). In contrast, fascia lata autograft was preferred in superior capsule reconstruction (9 studies). Post-operative management was well reported in the majority of studies; 70 described a progressive rehabilitation programme.

The pre-operative management of patients was described in 28 studies, the majority of these documented a minimum of 6 months of failed conservative therapy. All surgical procedures were performed in the beach chair or lateral decubitus position. In the case of bridging repairs and superior capsule reconstruction, allografts, autografts, xenografts and synthetic grafts were used (Tables 2, 3, 4, 5 and 6).

In all cases of debridement and balloon spacer insertion, pendular and passive range of motion exercises were commenced within 4 weeks, with the majority initiating range of motion exercises from the first day post-op. Sling use varied, it was used for a maximum of 3–4 weeks following these two procedures. In contrast, following arthroscopic PCR, SCR or graft interposition, patients were immobilised in a sling for up to 6 weeks followed by passive and active range of motion exercises. Full strengthening work may not begin for 6 months.

#### Partial cuff repair

See Table 2.

#### Graft interposition

See Table 3.

#### Superior capsule reconstruction

See Table 4.

#### Arthroscopic debridement

See Table 5.

#### Balloon spacer

See Table 6.

### Function scores and patient-reported outcome measures

In total, 49 studies reported pre-operative and post-operative Constant-Murley scores. The ASES was the next most reported score (32 studies) followed by the UCLA Shoulder Score (15 studies).

In all studies, there was an improvement in post-operative PROMs data compared to pre-operative scores. Synthesis of the difference in pre-operative and post-operative PROMs scores was possible across 51 studies. The pooled standardised mean difference for each implant is given in Table 7. Data were available at 1 year and 2 years for all 5 procedures. Three-year data were available for balloon spacers only and 5-year data for arthroscopic partial cuff repair and arthroscopic debridement. The number of studies contributing to the pooled SMD is shown in brackets. The greatest number was available for partial cuff repair and superior capsule reconstruction. A study by Vad et al. demonstrated a very high risk of bias and an SMD far greater than similar studies [81]. Results were pooled with and without inclusion of this study and omitted in the results given in Table 7. The full series of forest plots are available in the Appendix.

**Table 7:** SMDs in the difference in pre-operative and post-operative PROMs scores. Number of articles pooled in brackets

Procedure	1 year	2 years	3 years	5 years
Partial cuff repair	3.18 (8)	3.92 (10)		3.42 (6)
Graft interposition	3.58 (7)	5.00 (3)		
Superior capsule recon	3.12 (10)	4.04 (9)		
Arthro. debridement	4.48 (5)	2.63 (4)		3.21 (2)
Balloon spacer	1.36 (2)	4.53 (3)	1.66 (2)	

**Fig. 2:**
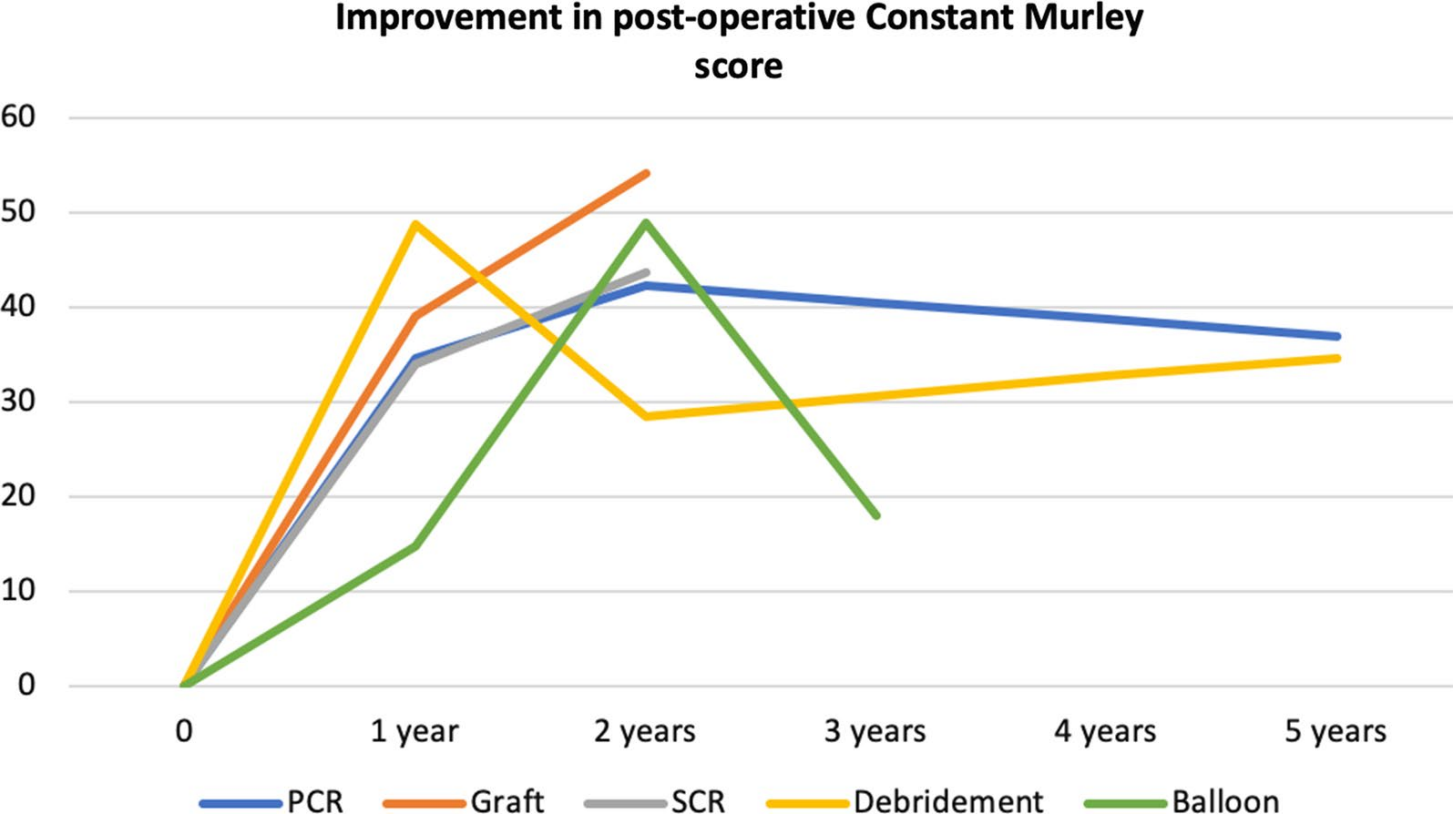
Standardised mean difference for each procedure, transformed to the Constant-Murley score

The SMD was re-expressed as a Constant-Murley score (Fig. 2); the mean standard deviation for the pre-operative Constant-Murley score was 10.9.

### Secondary outcomes

The quality of reporting of revision procedures, retear rates and complications was mixed (results summarised in Tables 8 and 9 and the Appendix). Post-operative imaging was performed to a different extent across the studies. This may include a routine MRI or ultrasound (US) in all cases, a proportion of cases, post-operative imaging only when the patient was symptomatic or not at all.

**Table 8:** Revision procedures, retears and infections following PCR, graft interposition and SCR

Procedure	No. of shoulders	Revision procedures for failure of procedure (%)	Post-op imaging performed	Retears	Infections
Partial cuff repair	733	2.9	170	45.3%	0.55%
Graft interposition	526	1.9	302	21.2%	0.39%
Superior capsule reconstruction	732	3.0	469	21.3%	0.14%

**Table 9:** Revision procedures and infections following arthroscopic debridement and balloon spacer

Procedure	No. of shoulders	Revision procedures for failure of procedure (%)	Infections
Arthroscopic debridement	438	2.9	0.2%
Balloon spacer	276	7.2	0%

Six studies reporting outcomes of PCR performed an MRI or US in all shoulders. Of 170 shoulders, 77 showed evidence of a retear (45%). Following graft interposition, nine studies reported post-operative imaging in all cases and further 6 studies scanned a proportion of patients. In total, 302 shoulders were scanned and 64 tears of the graft or tendon were identified (21%). After SCR, 12 studies reported imaging for all shoulders, and a further 5 scanned a majority of shoulders for a total of 469; 100 retears were found (21%).

Revision procedures for failure or symptom recurrence were poorly documented. Some studies may have only reported on patients that achieved the documented minimum follow-up period, resulting in an underestimation of the true revision rate. The greatest proportion of revision procedures was performed after balloon spacers (7.2%). The fewest revisions were reported following graft interposition (1.9%).

Reporting of additional complications was sparse; these are documented in the Appendix. Polacek et al. reported three cases of acute porcine dermal xenograft rejection which required urgent revision [71]. Two cases of donor site morbidity were reported in two studies where fascia lata autograft was used for a SCR, and one required revision surgery at the donor site [72, 73]. Twenty studies reported VAS scores. In all studies, there was improvement in the post-operative pain scores. The limited data on pain scores preclude meaningful comparison between techniques.

### Risk of bias

A risk-of-bias assessment of the included studies was performed according to the MINORS instrument and is summarised in Table 10 [11]. The cohort studies were generally of low quality. In order to maximise the opportunity for comparison between procedures, we extracted each relevant arm of the comparison separately and treated them as individual series. Consequently, the abbreviated 7-point MINORS criteria for non-comparative studies were calculated for all included studies. The results are summarised in Table 10. The full tables, including the results of the extended MINORS instrument for comparative studies, are available in the Appendix. For each procedure the mean risk of bias score across all seven domains was calculated.

**Table 10:** Risk-of-bias scores

Procedure	Clearly state aim	Inclusion of consecutive patients	Prospective data collection	Unbiased endpoint assessment	Follow-up appropriate	Loss to f/u < 5%	Prospective calculation sample size	Mean score
Partial cuff repair	2	1.46	1.62	2	2	1.54	0.23	**1.55**
Graft interposition	1.72	1.78	1.83	2	2	1.83	0	**1.60**
Superior capsule recon	2	1.86	1.62	2	2	1.67	0.19	**1.62**
Arthro. debridement	2	1.15	1.62	2	2	1.46	0	**1.46**
Balloon spacer	2	1.3	2	2	2	0.7	0	**1.43**

The greatest risk of bias was seen in studies on balloon spacers. These frequently described significant numbers lost to follow-up, and similar to all studies in this review, outcome scores were only presented for patients that completed follow-up.

## Discussion

This systematic review and meta-analysis compared common surgical techniques for the management of large and massive irreparable rotator cuff tears. Management of this pathology in the absence of glenohumeral arthritis is challenging. This work demonstrates that arthroscopic debridement, arthroscopic PCR, SCR, subacromial balloon spacers and graft interposition all lead to improved shoulder scores at early- to mid-term follow-up. It is encouraging for surgeons and patients that there are several potential surgical strategies which lead to improvements in shoulder function. However, we show that clinical improvement may decline over time for arthroscopic debridement, PCR and balloon spacers, and this is most apparent for balloon spacers. Across the included case series, the improvement in shoulder scores for PCR was comparable with graft interposition and SCR. Despite the additional cost of graft augmentation and the additional expertise required for SCR, they may not provide further clinical benefit in the early to mid-term period compared to PCR.

The number of studies and participants available for synthesis at each time point varied. Three hundred and ninety participants from 10 studies were included in the synthesis of SCR 12-month outcomes compared to 50 in two studies for arthroscopic debridement at 5 years. We required a minimum post-operative duration of all patients within a series to be included in the quantitative synthesis. The greatest volume of evidence was available for graft interposition, partial cuff repair and superior capsule reconstruction. The pooled PROMs scores for each of these procedures were similar up to 2 years.

The results of arthroscopic debridement and subacromial spacers should be interpreted with caution. The trajectory of improvement in the post-operative scores for arthroscopic debridement shown in Fig. 2 demonstrates a decline in scores at a minimum of 24 months followed by an improvement at 5 years. In the 5-year group, there were two studies with a moderate to high risk of bias and a total of only 52 patients. The studies reporting on subacromial spacers had a higher failure rate and loss to follow-up. A randomised trial comparing balloon spacer to arthroscopic debridement demonstrated superior shoulder scores following arthroscopic debridement at 12 months [94]. A further trial comparing subacromial spacers to PCR demonstrated non-inferiority of subacromial spacers at 12 months [95]. However, despite improvement in the first 12–24 months after PCR and subacromial spacers, our work shows a decline in PROMs after this point, which is most prominent following subacromial spacers.

Reverse shoulder arthroplasty (RSA) was not included in this review. The use of RSA is rapidly expanding. However, they are performed infrequently in the absence of glenohumeral osteoarthritis, particularly in younger patients. RSA can be effective at mid- to long-term follow-up in patients with massive tears, with or without arthritic disease [96–98]. Ernstbrunner et al. reported that improvement in shoulder scores may be sustained over 10 years in the presence of massive tears; however, survivorship was 85% at 5 years and 70% at 15 years [97]. Other series demonstrate a sustained improvement in shoulder scores and a lower incidence of complications in the absence of glenohumeral osteoarthritis; however, these remain a concern [98, 99]. The National Joint Registry of England, Wales and Northern Ireland report a risk of revision of 3.85% at 8 years in all patients who receive a RSA, with a median patient age of 76 years [100]. Current evidence would suggest caution and shared decision-making when considering RSA in younger patients without osteoarthritis.

Latissimus dorsi and trapezius tendon transfers show encouraging results in specific patient groups. Recent reviews report large increases in shoulder scores across several series [16, 101, 102]; improved shoulder function may persist over 10 years[103]. A comparative study by Cavalier demonstrated an inferior Constant score at 12 months with latissimus dorsi tendon transfer compared to PCR and RSA, and a randomised trial of 42 patients reported inferior functional scores for LDTT compared to SCR [16, 68].

This review included a broad and comprehensive search strategy and included a large number of patients not included in earlier work. Previous systematic reviews have attempted to compare procedures for irreparable or massive rotator cuff tears [104, 105]. We have captured multiple studies not included in previous reviews. This is the first study to include a quantitative synthesis across a large number of studies accounting for different outcome scores.

This study has limitations. The quality of included studies varied, and reliable conclusions cannot be made about the comparative effectiveness of each procedure. Although further well-constructed and appropriately powered randomised trails comparing these techniques are warranted, they are practically challenging given the varied nature of tendon reparability and tendon quality in patients with large and massive tears. Despite an accepted definition of an irreparable tendon as one that cannot be brought back to the tendon footprint without undue tension, intra-operative assessments may differ and the techniques used to bring the tendon back to the footprint, such as interval slide and marginal convergence, are not routinely described—the decision of whether a tendon can be brought to the greater tuberosity may influence outcomes in borderline cases. We did not classify studies according to pre-operative management. This was poorly reported; dedicated anterior deltoid strengthening work can improve shoulder function in patients with irreparable cuff tears [106]. We did investigate the change in shoulder scores, rather than post-operative score alone which would account for any large differences in pre-operative function.

## Conclusion

In adult patients with large and massive irreparable rotator cuff tears arthroscopic debridement, arthroscopic PCR, SCR, subacromial balloon spacers and graft interposition lead to improved early- and mid-term patient-reported outcomes and functional scores. Retear rates are 21% for SCR, 21% for graft interposition, and 45% for arthroscopic PCR. Mid- to long-term follow-up is necessary to further investigate whether there is a significant decline in shoulder function with time.

## Appendix

### Complete results tables: shoulder scores

#### Partial cuff repair

See Table 11.

**Table 11:** Partial cuff repair—included studies

Author and Date	No. of shoulders at f/u—PROMS	Study results pooled	Functional/PROMs outcomes pooled	Pre-op scores	12-month scores	24-month scores	X-month scores (specified minimum duration in months)
Baverel [13]	26	Yes	Constant, ASES, SSV, SST	36.7 (16.6) 33.5 (16.4) 37.5 (15.4) 3.5 (2.6)	64.8 (13.7) 78.3 (19.3) 73.3 (17.5) 8.3 (2.4)		
Berth [14]	21	Yes	Constant, QuickDASH	45.9 (9.2) 64.6 (11.9)	79.4 (17.5) 16 (16.1)	72.8 (16) 23.8 (16.8)	
Besnard [15]	20	Yes	Constant	31 (9.2)		77.1 (13)	72.8 (14.4)–85 m
Cavalier [16]	67	No	Constant, ASES, SSV	51 (n/r) 34 (n/r) 42 (n/r)	72 (n/r) 77.9 (n/r) 73 (n/r)		
Chen [17]	37	Yes	Constant,	45.95 (20.56)		78.59 (14.29)	
Cuff [18]	28	Yes	ASES, SST	46.6 (6.9) 5.6 (1.3)	84.2 (4.1) 10.2 (0.8)		79.3 (7.8)–60 m 9.1 (1.4)–60 m
Di Benedetto [19]	31	Yes	Constant	46.5 (11.5)			70.82 (14.66)–79 m
Farazdaghi [20]	14	No	ASES Penn	41.2 (10.1) 42.0 (12.5) Not PCR group alone			70.6 (32.9)–60 m 71.1 (30.4)–60 m
Franceschi [21]	34	Yes	UCLA	8.6 (4.1)		32.2 (3.6)	28.8 (4.2)–60 m
Galasso [22]	95	Yes	Constant, SST	39.1 (8.4) n/r		76.3 (9.7) 9.1 (2.2)	
Greiner [23]	20	No	Constant, DASH	50.7 (n/r) n/r		82.7 (8.4) 7.8 (11.1)	
Heuberer [24]	41	No	Constant QuickDASH	In graph form only	67.5 (9.9) 20.5 (14.4)		
Holtby [25]	73	No	Constant, ASES	44.03 (n/r) 42.69 (n/r)		73.73 (n/r) 71.42 (n/r)	
Jeong [26]	33	Yes	UCLA, ASES, SSV,	15.1 (3) 40.9 (8.9) 38.4 (8.4)			27.5 (2.8)–60 m 82.6 (7.1)–60 m 83.1 (6.8)–60 m
Kim [27]	27	Yes	Constant, SST	43.6 (7.9) 5.1 (1.2)		74.1 (10.6) 8.8 (2.1)	
Kim [28]	19	Yes	UCLA, ASES, SST	15.4 (2.9) 40.3 (9.3) 4.7 (1.4)		28.4 (3.2) 85.6 (8.3) 8.8 (1.8)	
Lee [29]	42	Yes	UCLA, Constant	20.5 (4.2) 41.2 (6.7)		30.9 (2.3) 88.8 (7.9)	
Malahias [30]	16	Yes	Constant, ASES	41.7 (15.6) 51 (16.5)	69.6 (19.7) 79.8 (18.8)		
Mori [31]	48	No	UCLA, Constant, ASES	13.7 (3.1) 36.3 (9.9) 41.8 (11.3)	27.3 (6.1) 72.9 (16.8) 84.2 (19.7)		
Moser [32]	11	No	SPADI	Collected but not reported		29.5 (n/r)	
Pandey [33]	13	Yes	Constant, OSS	43.1 (3.9) 17.8 (3.6)		70.8 (5.3) 37.1 (2.4)	
Park [34]	37	Yes	Constant, ASES, KSS	78 (11.6) 51.5 (22.7) 62.2 (14.1)	87.4 (8.3) 72.8 (19.2) 77.4 (12.6)	91.0 (7.4) 78.5 (18.5) 82.2 (13.2)	
Paribelli [35]	20	Yes	UCLA	7.3 (2.5)	30.3 (4.2)		
Porcellini [36]	67	Yes	Constant, SST	44 (14.1) 4.6 (2.3)			73 (11.9)–60 m 9.0 (1.8) –60 m
Shon [37]	31	Yes	ASES, SST	41.97 (15.08) 3.61 (2.58)	76.37 (17.01) 6.33 (2.58)	73.78 (21.55) 6.07 (3.4)	
Wellmann [38]	38	No	Constant	56 (n/r)	71 (n/r)		

PCR–partial cuff repair, LDTT—latissimus dorsi tendon transfer, Constant—Constant-Murley Score, QuickDASH–Disabilities of the Arm, Shoulder and Hand Score, ASES—American Shoulder and Elbow Score, SSV—Subjective Shoulder Value, SST—Simple Shoulder Test, SPADI—Shoulder Pain and Disability Index, UCLA—The University of California at Los Angeles Shoulder Score, KSS—Korean Shoulder Score, OSS—Oxford Shoulder Score

#### Graft interposition

See Table 12.

**Table 12:** Graft interposition—included studies

Author and Date	No. of shoulders at f/u	Study results pooled	Outcomes measured	Pre-op scores (SD)	12-month scores (SD)	24-month scores (SD)	X-month scores—specified duration (SD)
Audenaert [39]	39	No	Constant	25.7 (n/r)		72.1 (n/r)	
Badhe [40]	10	Yes	Constant	41.5 (17.5)	62.5 (14.2)		62.2 (14.5)–36 m
Bond [41]	16	Yes	Constant UCLA	53.9 (10.6) 18.4 (4.2)	84 (8.9) 30.4 (4.0)		
Dukan [42]	23	Yes	Constant	34.7 (7.8)		78.3 (16.5)	
Gupta [43]	24	No	Constant	66.6 (n/r)		88.7 (17.7)	
Gupta [44]	27	No	Constant	62.7 (n/r)		91.8 (13.3)	
Kim [45]	24	No	UCLA, ASES, SST	17 (n/r) 50 (n/r)		30 (n/r) 83 (n/r)	
Kokkalis [46]	21	Yes	ASES	25.2 (6.78)	74.4 (16.13)	14 pts 24 months (pre-op 25.5, 6.38) (72.4, 14.23)	
Modi [47]	61	No	OSS	26 median (n/r)	42 median (n/r)		
Mori [31]	48	No	UCLA, Constant, ASES	14.3 (2.9) 37.4 (8.1) 40.8 (13)	28.6 (4.3) 73.6 (6.6) 84.9 (8.1)		
Nada [48]	21	No	Constant	46.7 (n/r)			36 months–84.5 (n/r)
Pandey [33]	13	Yes	Constant, OSS	41.2 (3.1) 14.9 (3.5)		83.9 (6) 43.9 (2.4)	
Petrie [49]	31	No	OSS (old score)	46.7 (n/r)		30.6 (n/r)	
Rhee [50]	31	No	UCLA, Constant	12.5 (n/r) 48.4 (n/r)		31.1 (n/r) 81.8 (n/r)	
Rhee [51]	24	Yes	ASES, QuickDASH,	45.4 (19.1) 50 (17.9)	81.6 (17.6) 14.2 (20)		
Sano [52]	14	Yes	JOA	54.6 (9.3)	83.1 (7.5)		
Dimitrios [53]	68	Yes	Constant	32.5 (8.74)	88.7 (7.44)		
Wong [54]	45	No	UCLA	18.4 (n/r)		27.5 (n/r)	

JOA—Japanese Orthopaedic Association Score

#### Superior capsule reconstruction

See Table 13.

**Table 13:** Superior capsule reconstruction—included studies

Author and Date	No. of shoulders at f/u—PROMS	Study results pooled	Functional/PROMs outcomes measured	Pre-op scores	12-month scores	24-month scores	X-month scores (specified)
Alarcon [55]	31	No	Constant	36.0 (n/r)		78.7 (n/r)	
Barth [56]	24	Yes	Constant, ASES, SST	50 (13) 45 (19) 9 (2)		77 (10) 80 (15) 8 (3)	
Burkhart [57]	41	Yes	ASES, SSV	52 (3) 39 (n/r)	90 (1) 88 (n/r)	89 (2) 83 (n/r)	
Denard [58]	59	Yes	ASES, SSV,	43.6 (18.6) 35.0 (19.9)	77.5 (22) 76.3 (25.2)		
Ferrando [59]	52	Yes	ASES, SSV	41 (19) 39 (17)	86 (16) 80 (18)	90 (9) 80 (11)	
Greiner [23]	20	No	Constant, DASH	49.7 (n/r) n/r		77.1 (10.5) 15.6 (15.4)	
Kim [60]	45	No	Constant, ASES	64.9 (10.9) 60.9 (12.7)	80.0 (9.4) 82.2 (9.2)	80.0 (9.1) 82.7 (9.3)	Healed and unhealed reported separately throughout paper
Kocaoglu [61]	26	Yes	ASES QuickDASH	48.5 (15.5) 53.6 (15.2)	82.6 (15) 12.5 (5)		
LaBelle [62]	28	Yes	SST, ASES	21.6 (17.6) 28.3 (10.1)		66.6 (22.6) 68.2 (19.2)	
Lacheta [63]	21	No	ASES, QuickDASH	54 (n/r) 37.6 (n/r)		83.9 (n/r) 16.2 (n/r)	
Lee and Min [64]	36	No	Constant, ASES	56.3 (8.8) 50.9 (8.9)	84.3 (4.5) 85.1 (4.4)		Healed and unhealed reported separately throughout paper
Lim [65]	31	Yes	Constant, ASES	51.7 (13.9) 54.4 (17.9)	63.7 (8.1) 73.7 (10.8)		
Mihata [5]	24	Yes	ASES, JOA	23.5 (14.4) 48.3 (13)		92.9 (11.3) 92.6 (9)	
Okamura [66]	35	No	ASES	42.4 (n/r)		63.2 (n/r)	
Ohta [67]	35	Yes	UCLA, JOA	15.3 (3.77) 62.3 (9.49)	30.1 (3.11) 84.6 (5.66)		
Ozturk [68]	20	Yes	Constant, ASES	36.6 (12.5) 23.2 (12.7)		81.1 (11.3) 81.7 (12.3)	
Pashuck [69]	14	Yes	ASES	55 (17)	83.3 (16)	86.5 (9)	
Pennington [70]	88	Yes	ASES	52.2 (19.3)	81.56 (10.21)	85.3 (n/r)	
Polacek [71]	20	Yes	SPADI	51.3 (19.2)	10.4 (8.8)		
Polacek [72]	24	Yes	SPADI	59.0 (19.4)	9.7 (12.3)		
Takayama [73]	46	Yes	ASES	52.4 (12.6)		86.1 (13.8)	

#### Arthroscopic debridement

See Table 14.

**Table 14:** Arthroscopic debridement—included studies

Author and Date	No. of shoulders at f/u—PROMS	Study results pooled	Functional/PROMs outcomes measured	Pre-op scores	12-month scores	24-month scores	X-month scores (specified)
Berth [14]	21	Yes	Constant,	37 (13.6)	61.3 (19.9)	24.2 m 50.4 (15.3)	
			QuickDASH	69.6 (10.5)	29.7 (19.7)		35.3 (18.6)
Blanke [74]	59	No	Constant	33.9 (range only)		54.5 (range only)	
Boileau [75]	72	Yes	Constant	46.3 (11.9)		66.5 (16.3)	
Cavalier [16]	67	No	Constant, ASES, SSV	52 (n/r) 30.5 (n/r) 43(n/r)	62 (n/r) 59.9 (n/r) 65 (n/r)		
Franceschi [21]	34	Yes	UCLA	7.6 (2.6)		23.2 (2.8)	21.4 (3.7)–60 m
Heuberer [24]	41	No	Constant QuickDASH	In graph form only	65.8 (14.7) 24.1 (20.6)		
Klinger [76]	33	No	Constant	37 (n/r)		67 (n/r)	
Lee [77]	32	No	Constant, UCLA	47.6 (n/r) 15.4 (n/r)		70.4 (n/r) 27.1 (n/r)	
Mirzaee [78]	12	Yes	UCLA	9.2 (0.8)	27.5 (0.8)		
Park [79]	16	Yes	UCLA, Constant	10.3 (2.7) 39.6 (7.6)	26.8 (5.4) 58.8 (9.7)		28.3 (4.6)–60 m 60.3 (10.2)–60 m
Scheibel [80]	22	No	Constant	65.9 (n/r)	90.6 (n/r)		
Vad [81]	32	Yes	Shoulder rating questionnaire	42.3 (1.4)		81.4 (1.3)	
Veado [82]	12	Yes	UCLA	14.9 (4.6)	29.9 (3.2)	23.7 (3.3)	
Verhelst [83]	32	Yes	Constant	34.9 (11.6)	84 (11.6)		

#### Balloon spacer

See Table 15.

**Table 15:** Balloon spacer—included studies

Author and Date	No. of shoulders at f/u—PROMS	Study results pooled	Functional/PROMs outcomes measured	Pre-op scores	12-month scores	24-month scores	X-month scores (specified)
Basat [84]	12	Yes	Constant	25.8 (5.31)		75.4 (6.05)	
Bakti [85]	26	No	OSS—in graph form only				
Deranlot [86]	39	Yes	Constant	40 (14.6)	59 (13.7)		64 (3 year) (13.6)
Familiari [87]	51	Yes	Constant	27 (7.4)		77 (15)	
Gervasi [88]	15	No	Constant, ASES	31.9 (n/r) 24.5 (n/r)	69.8 (n/r) 76 (n/r)		
						72.5 (n/r)	
Gervasi [89]	40	Yes	Constant, ASES	28.6 (11.6) 24.4 (11.8)		67.9 (16.7) 84.2 (21.0)	
Iban [90]	10	No	Constant, SST, QuickDASH	35 median (n/r) 3 median (n/r) 37 median (n/r)	62.5 (n/r) 5 (n/r) 30 (n/r)	53.5 (n/r) 6 (n/r) 27.5 (n/r)	
Lorente [91]	15	No	Constant	30 (range)	47 (range)		
Maman [92]	42	No	Constant	36 (n/r)	65.8 (n/r)	70.8 (n/r)	
Senekovic [93]	18	Yes	Constant	33.41(13.34) 20 pts	60.46 (22.98) 18 pts		65.42 (25.23)–36 m 16 pts

## Forest plots

### PCR 12 months


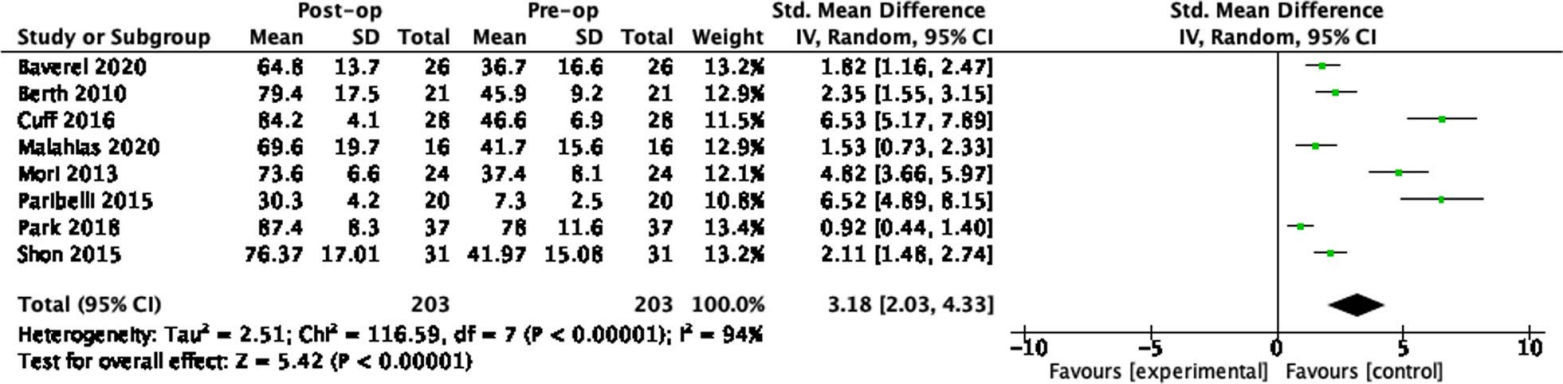


### PCR 24 months


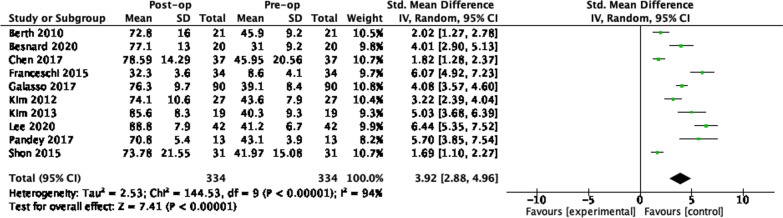


### PCR 5 years


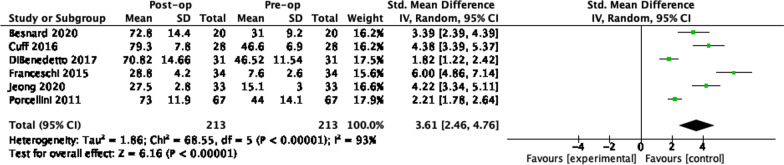


### Graft interposition 12 months


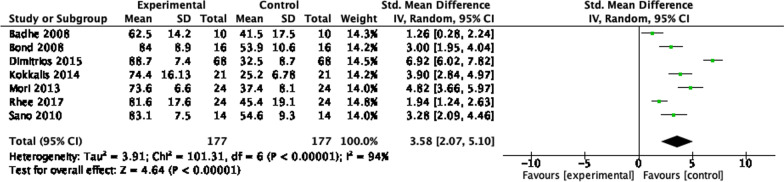


### Graft interposition 24 months


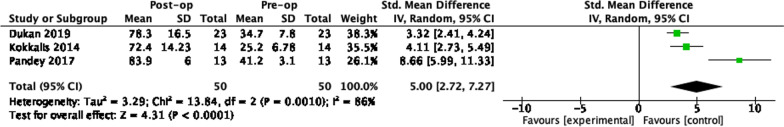


### SCR 12 months


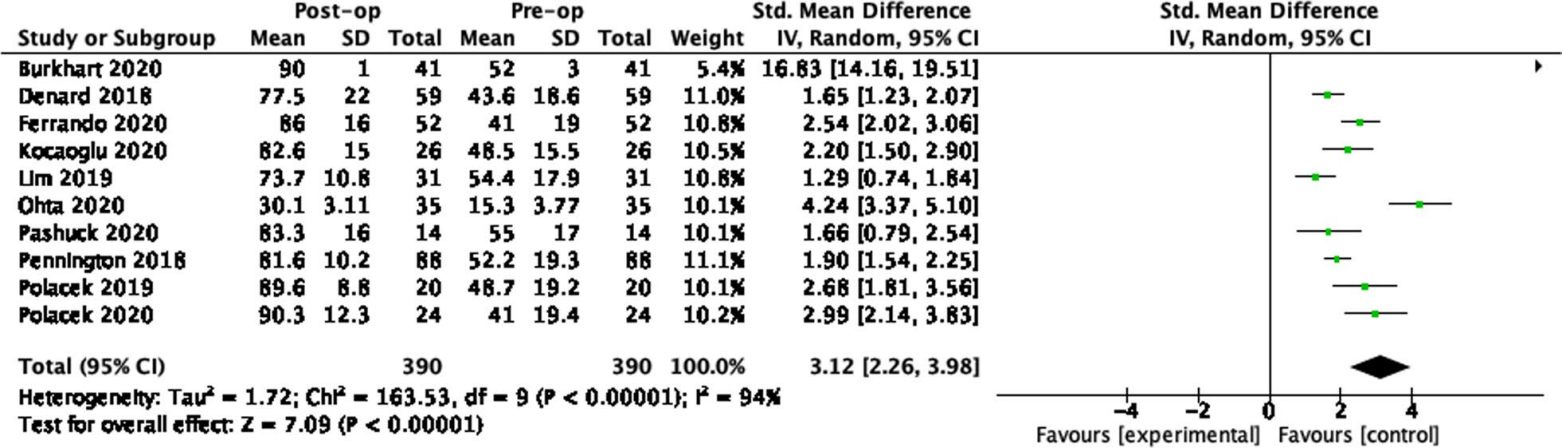


### SCR 24 months


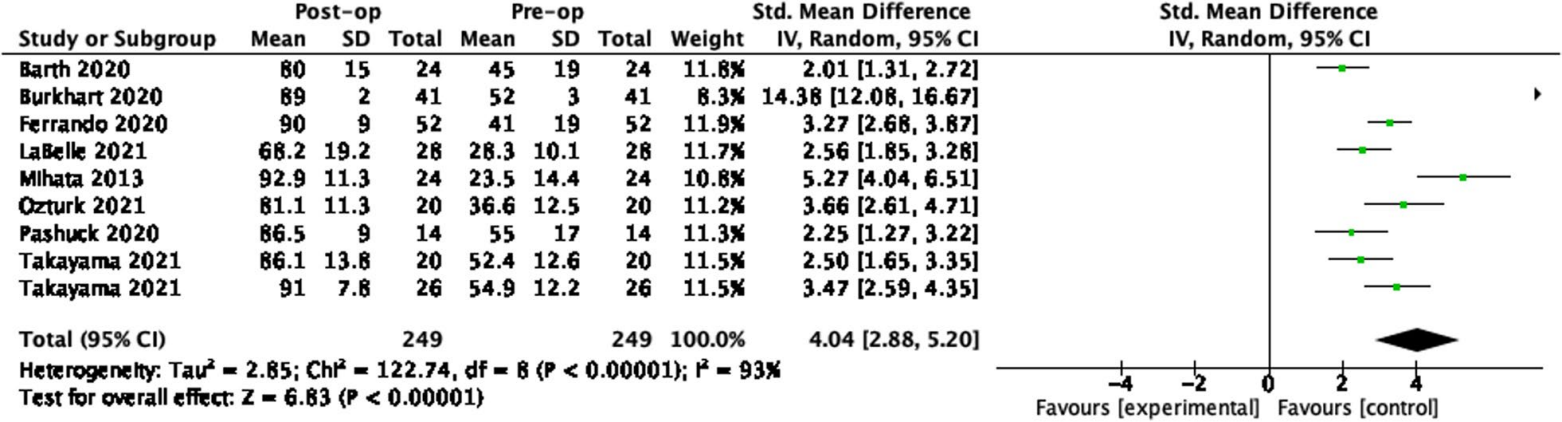


### Arthroscopic debridement 12 months


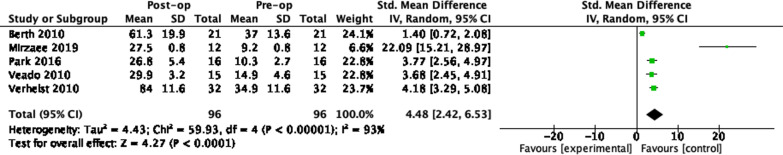


### Arthroscopic debridement 24 months


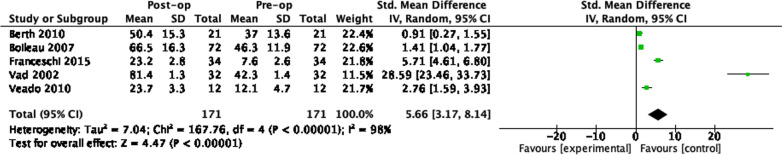



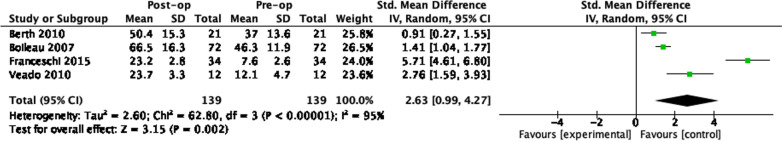


### Arthroscopic debridement 5 years


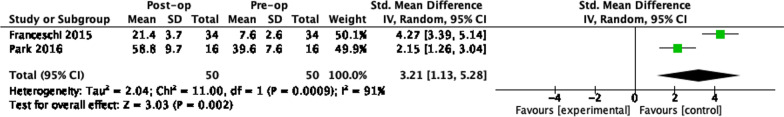


### Balloon 12 months


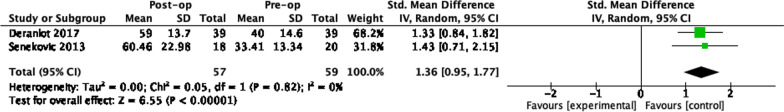


### Balloon 24 months


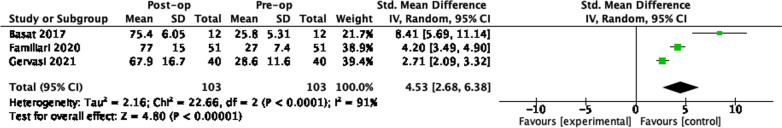


### Balloon 3 years


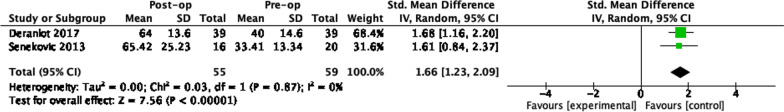


## Complications

### Partial cuff repair

**Table:** 

Author and Date	Number of shoulders in PCR group for which f/u data available	No. of revision procedures for failure procedure	Post-op imaging performed—no. of patients	Number retears on imaging	Infections	Additional complications
Baverel [13]	26	0	0	n/a	0	0
Berth [14]	21	1	21	11	0	0
Besnard [15]	20	4	0	n/a	n/r	n/r
Cavalier [16]	61	0	0	n/a	1	1 anchor migration
Chen [17]	37	0	Unknown	Unknown	n/r	n/r
Cuff [18]	28	3	0	n/a	0	0
DiBenedetto [19]	31	0	0	n/a	n/r	n/r
Farazdaghi [20]	14	4	0	n/a	0	0
Franceschi [21]	34	n/r	0	n/a	n/r	n/r
Galasso [22]	95	8	0	n/a	n/r	n/r
Greiner [23]	20	0	0	n/a	0	0
Heuberer [24]	22	1	Unknown	Unknown	2 (washouts)	0
Holtby [25]	73	n/r	0	n/a	n/r	n/r
Jeong [26]	33	0	33	28	n/r	n/r
Kim [27]	27	0	0	n/r	n/r	n/r
Kim [28]	19	0	0	n/r	n/r	n/r
Lee [29]	42	0	42	10	0	0
Malahias [30]	16	0	0	n/a	1	0
Mori [31]	24	0	24	10	0	0
Moser [32]	11	0	0	n/a	0	0
Pandey [33]	13	0	13	4	0	0
Park [34]	37	0	37	14	0	0
Paribelli [35]	20	0	0	n/a	n/r	n/r
Porcellini [36]	67	0	0	n/a	0	2 stiffness (capsular release)
Shon [37]	31	0	0	n/a	n/r	n/r
Wellmann [38]	38	0	0	n/a	n/r	n/r

### Graft interposition

**Table:** 

Author and Date	Number of shoulders in graft group for which f/u data available	No. of revision procedures	Post-op imaging performed—no. of patients	Number of retears on imaging	Infections	Additional complications
Audernaert [39]	39	0	39	4	0	0
Badhe [40]	10	0	10	2	0	0
Bond [41]	16	0	16	3	0	0
Dukan [42]	23	5	18	9	0	2 intraop partial glenoid fractures
Gupta [43]	24	0	19	5	0	0
Gupta [44]	27	0	22	6	0	0
Kim [45]	24	0	24	5	0	0
Kokkalis [46]	21	0	0	n/a	0	0
Modi [47]	61	1	14	2	1 (washout)	0
Mori [31]	24	0	24	5	0	0
Nada [48]	21	1	21	0	0	0
Pandey [33]	13	0	13	1	0	0
Petrie [49]	31	2	0	n/a	0	0
Rhee [50]	31	1	14	5	0	0
Rhee [51]	24	0	24	13	n/r	n/r
Sano [52]	14	0	14	1	0	0
Dimitrios [53]	68	0	30	3	0	0
Wong [54]	45	0	0	n/a	1 (washout)	0

### Superior capsule reconstruction

**Table:** 

Author and Date	Number of shoulders in SCR group for which f/u data available	No. of revision procedures	Post-op imaging performed—no. of patients	Number of retears on imaging	Infections	Additional complications
Alarcon [55]	31	0	29	4	0	1 haematoma
Barth [56]	24	0	24	2	n/r	n/r
Burkhart [57]	41	2 (post-falls)	26	4	0	1 stroke 1 tear biceps tenodesis
Denard [58]	59	0	20	11	1	1 biceps pain
Ferrando [59]	56	4	56	14	n/r	n/r
Greiner [23]	20	0	20	1	n/r	n/r
Kim [60]	45	0	45	6	0	1 skin dehiscence, 2 Popeye sign
Kocaoglu [61]	26	0	12	2	n/r	n/r
LaBelle [62]	35	4	21	13	n/r	n/r
Lacheta [63]	22	1	21	5	n/r	n/r
Lee and Min [64]	36	0	36	13	n/r	n/r
Lim [65]	31	0	31	9	0	1 PE
Mihata [5]	24	0	24	4	0	0
Okamura [66]	35	0	35	2	0	1 synovitis
Ohta [67]	35	1	35	7	0	1 severe synovitis 1 anchor dislodgement
Ozturk [68]	20	0	20	1	0	1 significant stiffness
Pashuck [69]	14	1	14	2	0	0
Pennington [70]	88	1	Only with Sx	-	n/r	n/r
Polacek [71]	20	5	Only with Sx	-	0	3 acute graft rejection
Polacek [72]	24	3 incl. 1 fascia lata site revision	Only with Sx	-	0	1 fascia lata muscle prolapse. 1 haematoma
Takayama [73]	46	0	Only with Sx	-		1 swelling at donor site 1 shoulder swelling

### Debridement

**Table:** 

Author and Date	Number of shoulders in debridement group for which f/u data available	No. of revision procedures	Infections	Additional complications
Berth [14]	21	1	0	0
Blanke [74]	59	0	0	0
Boileau [75]	72	3	1 (washout)	2 persistent pain. 1 shoulder stiffness, 2 GHJ OA, 3 pseudo paralysis
Cavalier [16]	26	0	0	0
Franceschi [21]	34	n/r	n/r	n/r
Heuberer [24]	23	1	0	0
Klinger [76]	41	0	0	3 pseudoparalysis 2 pain 1 stiffness. 2 GHJ OA. 1 humeral head migration
Lee [77]	32	4	n/r	n/r
Mirzaee [78]	12	0	0	2 GHJ OA
Park [79]	16	1	0	0
Scheibel [80]	23	1	0	1 haematoma
Vad [81]	32	0	n/r	n/r
Veado [82]	15	0	n/r	n/r
Verhelst [83]	32	2	n/r	n/r

### Balloon spacer

**Table:** 

Author and Date	Number of shoulders in balloon spacer group for which f/u data available	No. of revision procedures	Infections	Additional complications
Basat [84]	12	0	n/r	n/r
Bakti [85]	26	1	0	0
Deranlot [86]	39	1	0	0
Familiari [87]	51	6	0	0
Gervasi [88]	15	1	0	0
Gervasi [89]	40	3	0	0
Iban [90]	16	5	0	0
Lorente 2017 [91]	15	3	n/r	n/r
Maman [92]	42	0	n/r	n/r
Senekovic [93]	20	0	0	0

## Risk of bias

All arms of relevant non-comparative and comparative studies were treated as individual series. The risk-of-bias results for the abbreviated 7-point MINORS instrument for non-comparative studies were applied to all articles and presented here. The results of the extended MINORS instrument for comparative studies are presented in the final section of this document.

### PCR

**Table:** 

Author and Date	Clearly state aim	Inclusion of consecutive patients	Prospective data collection	Unbiased endpoint assessment	Follow-up appropriate	Loss to f/u < 5%	Prospective calculation of sample size
Baverel [13]	*2*	*2*	*2*	*2*	*2*	*2*	***0***
Berth [14]	*2*	**1**	*2*	*2*	*2*	*2*	***0***
Besnard [15]	*2*	*2*	**1**	*2*	*2*	***0***	***0***
Cavalier [16]	*2*	*2*	*2*	*2*	*2*	*2*	***0***
Chen [17]	*2*	**1**	**1**	*2*	*2*	**1**	***0***
Cuff [18]	*2*	**1**	**1**	*2*	*2*	**1**	***0***
DiBenedetto [19]	*2*	*2*	**1**	*2*	*2*	*2*	***0***
Farazdaghi [20]	*2*	**1**	**1**	*2*	*2*	**1**	*2*
Franceschi [21]	*2*	**1**	*2*	*2*	*2*	*2*	*2*
Galasso [22]	*2*	*2*	*2*	*2*	*2*	***0***	***0***
Greiner [23]	*2*	*2*	*2*	*2*	*2*	*2*	*2*
Heuberer [24]	*2*	**1**	*2*	*2*	*2*	***0***	***0***
Holtby [25]	*2*	*2*	*2*	*2*	*2*	*2*	***0***
Jeong [26]	*2*	**1**	**1**	*2*	*2*	**1**	***0***
Kim [27]	*2*	*2*	**1**	*2*	*2*	*2*	***0***
Kim [28]	*2*	*2*	**1**	*2*	*2*	*2*	***0***
Lee [29]	*2*	**1**	**1**	*2*	*2*	***0***	***0***
Malahias [30]	*2*	**1**	*2*	*2*	*2*	*2*	***0***
Mori [31]	*2*	*2*	*2*	*2*	*2*	*2*	***0***
Moser [32]	*2*	**1**	*2*	*2*	*2*	*2*	***0***
Pandey [33]	*2*	*2*	*2*	*2*	*2*	*2*	***0***
Park [34]	*2*	**1**	*2*	*2*	*2*	*2*	***0***
Paribelli [35]	*2*	**1**	*2*	*2*	*2*	*2*	***0***
Porcellini [36]	*2*	*2*	*2*	*2*	*2*	**1**	***0***
Shon [37]	*2*	**1**	**1**	*2*	*2*	*2*	***0***
Wellmann [38]	*2*	**1**	**1**	*2*	*2*	*2*	***0***

### Graft interposition

**Table:** 

Author and Date	Clearly state aim	Inclusion of consecutive patients	Prospective data collection	Unbiased endpoint assessment	Follow-up appropriate	Loss to f/u < 5%	Prospective calculation of sample size
Audernaert [39]	*2*	*2*	*2*	*2*	*2*	*2*	***0***
Badhe [40]	*2*	*2*	*2*	*2*	*2*	*2*	***0***
Bond [41]	*2*	*2*	*2*	*2*	*2*	*2*	***0***
Dukan [42]	*2*	*2*	*2*	*2*	*2*	**1**	***0***
Gupta [43]	**1**	*2*	*2*	*2*	*2*	*2*	***0***
Gupta [44]	*2*	*2*	*2*	*2*	*2*	*2*	***0***
Kim [45]	*2*	*2*	*2*	*2*	*2*	*2*	***0***
Kokkalis [46]	**1**	*2*	**1**	*2*	*2*	*2*	***0***
Modi [47]	*2*	*2*	*2*	*2*	*2*	*2*	***0***
Mori [31]	*2*	*2*	*2*	*2*	*2*	*2*	***0***
Nada [48]	**1**	***0***	*2*	*2*	*2*	*2*	***0***
Pandey [33]	*2*	*2*	*2*	*2*	*2*	*2*	***0***
Petrie [49]	*2*	*2*	*2*	*2*	*2*	**1**	***0***
Rhee [50]	*2*	**1**	*2*	*2*	*2*	**1**	***0***
Rhee [51]	**1**	*2*	*2*	*2*	*2*	*2*	***0***
Sano [52]	*2*	**1**	***0***	*2*	*2*	*2*	***0***
Varvitsiotis [53]	*2*	*2*	*2*	*2*	*2*	*2*	***0***
Wong [54]	**1**	*2*	*2*	*2*	*2*	*2*	***0***

### Superior capsule reconstruction

**Table:** 

Author and Date	Clearly state aim	Inclusion of consecutive patients	Prospective data collection	Unbiased endpoint assessment	Follow-up appropriate	Loss to f/u < 5%	Prospective calculation of sample size
Alarcon [55]	*2*	*2*	**1**	*2*	*2*	*2*	***0***
Barth [56]	*2*	*2*	**1**	*2*	*2*	**1**	***0***
Burkhart [57]	*2*	*2*	**1**	*2*	*2*	**1**	***0***
Denard [58]	*2*	**1**	*2*	*2*	*2*	**1**	***0***
Ferrando [59]	*2*	*2*	*2*	*2*	*2*	*2*	***0***
Greiner [23]	*2*	*2*	*2*	*2*	*2*	*2*	*2*
Kim [60]	*2*	*2*	**1**	*2*	*2*	**1**	***0***
Kocaoglu [61]	*2*	*2*	**1**	*2*	*2*	*2*	***0***
LaBelle [62]	*2*	*2*	**1**	*2*	*2*	**1**	***0***
Lacheta [63]	*2*	*2*	*2*	*2*	*2*	*2*	***0***
Lee and Min [64]	*2*	*2*	**1**	*2*	*2*	**1**	***0***
Lim [65]	*2*	*2*	*2*	*2*	*2*	*2*	***0***
Mihata [5]	*2*	*2*	*2*	*2*	*2*	*2*	***0***
Okamura [66]	*2*	*2*	*2*	*2*	*2*	*2*	*2*
Ohta [67]	*2*	**1**	**1**	*2*	*2*	**1**	***0***
Ozturk [68]	*2*	*2*	*2*	*2*	*2*	*2*	***0***
Pashuck [69]	*2*	**1**	*2*	*2*	*2*	*2*	***0***
Pennington [70]	*2*	*2*	*2*	*2*	*2*	*2*	***0***
Polacek [71]	*2*	*2*	*2*	*2*	*2*	*2*	***0***
Polacek [72]	*2*	*2*	*2*	*2*	*2*	*2*	***0***
Takayama [73]	*2*	*2*	*2*	*2*	*2*	*2*	***0***

### Balloon spacer

**Table:** 

Author and Date	Clearly state aim	Inclusion of consecutive patients	Prospective data collection	Unbiased endpoint assessment	Follow-up appropriate	Loss to f/u < 5%	Prospective calculation of sample size
Basat [84]	*2*	**1**	*2*	*2*	*2*	*2*	***0***
Batki [85]	*2*	**1**	*2*	*2*	*2*	***0***	***0***
Deranlot [86]	*2*	*2*	*2*	*2*	*2*	*2*	***0***
Familiari [87]	*2*	**1**	*2*	*2*	*2*	***0***	***0***
Gervasi [88]	*2*	**1**	*2*	*2*	*2*	***0***	***0***
Gervasi [89]	*2*	**1**	*2*	*2*	*2*	**1**	***0***
Iban [90]	*2*	*2*	*2*	*2*	*2*	***0***	***0***
Lorente [91]	*2*	*2*	*2*	*2*	*2*	**1**	***0***
Maman [92]	*2*	**1**	*2*	*2*	*2*	***0***	***0***
Senekovic [93]	*2*	**1**	*2*	*2*	*2*	**1**	***0***

### Debridement

**Table:** 

Author and Date	Clearly state aim	Inclusion of consecutive patients	Prospective data collection	Unbiased endpoint assessment	Follow-up appropriate	Loss to f/u < 5%	Prospective calculation of sample size
Berth [14]	*2*	**1**	*2*	*2*	*2*	*2*	***0***
Blanke [74]	*2*	**1**	**1**	*2*	*2*	*2*	***0***
Boileau [75]	*2*	**1**	**1**	*2*	*2*	*2*	***0***
Cavalier [16]	*2*	*2*	*2*	*2*	*2*	*2*	***0***
Franceschi [21]	*2*	**1**	*2*	*2*	*2*	*2*	***0***
Heuberer [24]	*2*	**1**	*2*	*2*	*2*	***0***	***0***
Klinger [76]	*2*	*2*	*2*	*2*	*2*	*2*	***0***
Lee [77]	*2*	*2*	*2*	*2*	*2*	*2*	***0***
Mirzaee [78]	*2*	*2*	*2*	*2*	*2*	*2*	***0***
Park [79]	*2*	**1**	**1**	*2*	*2*	**1**	***0***
Scheibel [80]	*2*	**1**	*2*	*2*	*2*	*2*	***0***
Vad [81]	*2*	***0***	***0***	*2*	*2*	***0***	***0***
Veado [82]	*2*	***0***	*2*	*2*	*2*	**1**	***0***
Verhelst [83]	*2*	**1**	*2*	*2*	*2*	**1**	***0***

## Additional full risk-of-bias table for comparative studies

**Table:** 

Author and Date	Comparisons	Clearly state aim	Inclusion of consecutive patients	Prospective data collection	Unbiased endpoint assessment	Follow-up appropriate	Loss to f/u < 5%	Prospective calculation of sample size	Adequate control group	Contemporary groups	Baseline equivalence of groups	Adequate statistical analyses
Baverel [13]	PCR vs LDTT	*2*	*2*	*2*	*2*	*2*	*2*	***0***	*2*	*2*	*2*	*2*
Berth [14]	PCR vs debridement	*2*	**1**	*2*	*2*	*2*	*2*	***0***	*2*	*2*	*2*	*2*
Besnard [15]	PCR vs complete	*2*	*2*	**1**	*2*	*2*	***0***	***0***	*2*	*2*	***0***	*2*
Cavalier [16]	PCR vs debridement	*2*	*2*	*2*	*2*	*2*	*2*	***0***	*2*	*2*	**1**	*2*
Farazdaghi [20]	PCR vs complete	*2*	**1**	**1**	*2*	*2*	**1**	*2*	*2*	*2*	***0***	*2*
Franceschi [21]	PCR vs debridement	*2*	**1**	*2*	*2*	*2*	*2*	*2*	*2*	*2*	*2*	*2*
Greiner [23]	PCR vs SCR	*2*	*2*	*2*	*2*	*2*	*2*	*2*	*2*	*2*	*2*	*2*
Heuberer [24]	PCR vs debridement	*2*	**1**	*2*	*2*	*2*	***0***	***0***	*2*	*2*	**1**	*2*
Holtby [25]	PCR vs complete	*2*	*2*	*2*	*2*	*2*	*2*	***0***	*2*	*2*	**1**	*2*
Jeong [26]	PCR vs complete	*2*	**1**	**1**	*2*	*2*	**1**	***0***	*2*	*2*	**1**	*2*
Kim [28]	PCR vs complete	*2*	*2*	*2*	*2*	*2*	*2*	***0***	*2*	*2*	*2*	*2*
Malahias [30]	PCR vs PCR and balloon	*2*	**1**	*2*	*2*	*2*	*2*	***0***	*2*	*2*	***0***	*2*
Mori [31]	PCR vs graft interposition	*2*	*2*	*2*	*2*	*2*	*2*	***0***	*2*	*2*	*2*	*2*
Moser [32] 2007	PCR vs complete vs debridement	*2*	**1**	*2*	*2*	*2*	*2*	***0***	*2*	*2*	*2*	*2*
Pandey [33]	PCR vs graft interposition	*2*	*2*	*2*	*2*	*2*	*2*	***0***	*2*	*2*	***0***	*2*
Park [34]	PCR vs augmentation	*2*	**1**	*2*	*2*	*2*	*2*	***0***	*2*	*2*	*2*	*2*
Paribelli [35]	PCR vs LDTT	*2*	**1**	*2*	*2*	*2*	*2*	***0***	*2*	*2*	*2*	*2*
Barth [56]	SCR vs complete vs augmentation	*2*	*2*	**1**	*2*	*2*	**1**	***0***	*2*	*2*	*2*	*2*
Kocaoglu [61]	SCR vs SCR and PCR	*2*	*2*	**1**	*2*	*2*	*2*	***0***	*2*	*2*	*2*	*2*
Okamura [66]	SCR **1** layer graft vs 3 layers	*2*	*2*	*2*	*2*	*2*	*2*	*2*	*2*	*2*	*2*	*2*
Ozturk [68]	SCR vs LDTT	*2*	*2*	*2*	*2*	*2*	*2*	***0***	*2*	*2*	*2*	*2*

## Search strategy

1. Rotator cuff/
2. Rotator Cuff Injuries/
3. 1 or 2
4. (massive or large or irreparable).ti,ab.
5. 3 and 4
6. ((massive or large or irreparable) adj3 (rotator cuff* adj3 (tear* or rupture* or injur*))).ti,ab.
7. ((massive or large or irreparable) adj3 (supraspinatus adj3 (tear* or rupture* or injur*))).ti,ab.
8. ((massive or large or irreparable) adj3 (infraspinatus adj3 (tear* or rupture* or injur*))).ti,ab.
9. ((massive or large or irreparable) adj3 (subscapularis adj3 (tear* or rupture* or injur*))).ti,ab.
10. ((massive or large or irreparable) adj3 (teres minor adj3 (tear* or rupture* or injur*))).ti,ab.
11. ((massive or large or irreparable) adj3 (posterosuperior adj3 (tear* or rupture* or injur*))).ti,ab.
12. 6 or 7 or 8 or 9 or 10 or 11
13. 5 or 12
14. Arthroscopy/
15. Debridement/
16. arthroscop*.ti,ab.
17. debridement*.ti,ab.
18. Superior capsul* reconstruction*.ti,ab.
19. capsul* reconstruction*.ti,ab.
20. Reconstructive Surgical Procedures/
21. Reconstruct*.ti,ab.
22. Repair*.ti,ab.
23. Tenotomy/
24. Tenodesis/
25. tenotom*.ti,ab.
26. tenodesis.ti,ab.
27. partial*.ti,ab.
28. 14 or 15 or 16 or 17 or 18 or 19 or 20 or 21 or 22 or 23 or 24 or 25 or 26 or 27
29. balloon*.ti,ab.
30. spacer*.ti,ab.
31. 29 or 30
32. graft*.ti,ab.
33. graftjacket.ti,ab.
34. patch*.ti,ab.
35. allografts/
36. autografts/
37. surgical mesh/
38. bioprosthesis/
39. tissue scaffolds/
40. extracellular matrix/
41. acellular dermis/
42. allograft*.ti,ab.
43. autograft*.ti,ab.
44. surgical mesh.ti,ab.
45. bioprosthe*.ti,ab.
46. tissue scaffold*.ti,ab.
47. extracellular matrix.ti,ab.
48. acellular dermal matrix.ti,ab.
49. bioartificial tendon*.ti,ab.
50. augment*.ti,ab.
51. "Zimmer Collagen Repair Patch".ti,ab.
52. "Permacol".ti,ab.
53. "TissueMend".ti,ab.
54. "BioBlanket".ti,ab.
55. "Conexa".ti,ab.
56. "AlloPatch".ti,ab.
57. "Shelhigh Encuff Patch".ti,ab.
58. "OrthADAPT".ti,ab.
59. "Restore".ti,ab.
60. "CuffPatch".ti,ab.
61. "Polytape".ti,ab.
62. "SportMesh".ti,ab.
63. "Arthelon".ti,ab.
64. "Gore-tex".ti,ab.
65. "BioFiber".ti,ab.
66. "STR Grafts".ti,ab.
67. "Lars Ligament".ti,ab.
68. "X-repair".ti,ab.
69. 32 or 33 or 34 or 35 or 36 or 37 or 38 or 39 or 40 or 41 or 42 or 43 or 44 or 45 or 46 or 47 or 48 or 49 or 50 or 51 or 52 or 53 or 54 or 55 or 56 or 57 or 58 or 59 or 60 or 61 or 62 or 63 or 64 or 65 or 66 or 67 or 68
70. 13 and 28
71. 13 and 31
72. 13 and 69
73. 70 or 71 or 72
74. exp animals/ not humans.sh.
75. 73 not 7472 13 and 69

73 70 or 71 or 72

74 exp animals/ not humans.sh.

75 73 not 74

## Data Availability

Further data are provided in the Appendix and full datasets can be provided by the corresponding author upon reasonable request.

## Abbreviations

CINAHL: Cumulative index to nursing and allied health literature
CT: Computed tomography
MRA: Magnetic resonance arthrogram
PCR: Partial cuff repair
SCR: Superior capsule reconstruction
PROM: Patient-reported outcome measures
PROSPERO: International prospective register of systematic reviews
PRISMA: Preferred Reporting Items for Systematic Reviews and Meta-Analyses
SLAP: Superior labral tear from anterior to posterior
ASES: American Shoulder and Elbow Score
OSS: Oxford Shoulder Score
UCLA: University of California in Los Angeles Shoulder Score
DASH: Disabilities of arm, shoulder and hand score
JOA: Japanese Orthopaedic Association Shoulder Score
MINORS: Methodological index for non-randomized studies
SMD: Standardised mean difference
MRI: Magnetic resonance imaging
LDTT: Latissimus dorsi tendon transfer
QuickDASH: Disabilities of the arm shoulder and hand score
SSV: Subjective shoulder value
SST: Simple shoulder test
SPADI: Shoulder pain and disability index
UCLA: The University of California at Los Angeles shoulder score
KSS: Korean Shoulder Score
SD: Standard deviation
US: Ultrasound
VAS: Visual analogue scale
RSA: Reverse shoulder arthroplasty

## References

[1] Linsell L, Dawson J, Zondervan K, Rose P, Randall T, Fitzpatrick R (2006). Prevalence and incidence of adults consulting for shoulder conditions in UK primary care; patterns of diagnosis and referral. Rheumatology.

[2] Woodmass JM, Wagner ER, Chang MJ, Welp KM, Elhassan BT, Higgins LD (2018). Arthroscopic treatment of massive posterosuperior rotator cuff tears. JBJS Rev.

[3] Burkhart SS (1991). Arthroscopic treatment of massive rotator cuff tears. Clin Orthop Relat Res.

[4] Mihata T, McGarry MH, Pirolo JM, Kinoshita M, Lee TQ (2012). Superior capsule reconstruction to restore superior stability in irreparable rotator cuff tears: a biomechanical cadaveric study. Am J Sports Med.

[5] Mihata T, Lee TQ, Watanabe C, Fukunishi K, Ohue M, Tsujimura T (2013). Clinical results of arthroscopic superior capsule reconstruction for irreparable rotator cuff tears. Arthrosc J Arthrosc Relat Surg.

[6] Catapano M, de SA D, Ekhtiari S, Lin A, Bedi A, Lesniak BP (2019). Arthroscopic superior capsular reconstruction for massive irreparable rotator cuff tears: a systematic review of modern literature. J Arthrosc Jt Surg..

[7] Omid R, Lee B (2013). Tendon transfers for irreparable rotator cuff tears. J Am Acad Orthop Surg.

[8] Castagna A, Garofalo R, Cesari E (2014). No prosthetic management of massive and irreparable rotator cuff tears. Shoulder Elb.

[9] Ek ETH, Neukom L, Catanzaro S, Gerber C, Hon F (2013). Reverse total shoulder arthroplasty for massive irreparable rotator cuff tears in patients younger than 65 years old : results after five to fifteen years. J Shoulder Elb Surg.

[10] Liberati A, Altman DG, Tetzlaff J, Mulrow C, Gøtzsche PC, Ioannidis JPA (2009). The PRISMA statement for reporting systematic reviews and meta-analyses of studies that evaluate health care interventions: explanation and elaboration. J Clin Epidemiol.

[11] Slim K, Nini E, Forestier D, Kwiatkowski F, Panis Y, Chipponi J (2003). Methodological index for non-randomized studies (minors): development and validation of a new instrument. ANZ J Surg.

[12] Higgins J, Li T, Deeks J. Chapter 6: Choosing effect measures and computing estimates of effect. In: cochrane handbook for systematic reviews of interventions. 2021. p Section 6-5-2-2.

[13] Baverel LP, Bonnevialle N, Joudet T, Valenti P, Kany J, Grimberg J (2021). Short-term outcomes of arthroscopic partial repair vs. latissimus dorsi tendon transfer in patients with massive and partially repairable rotator cuff tears. J Shoulder Elb Surg..

[14] Berth A, Neumann W, Awiszus F, Pap G (2010). Massive rotator cuff tears: functional outcome after debridement or arthroscopic partial repair. J Orthop Traumatol.

[15] Besnard M, Freychet B, Clechet J, Hannink G, Saffarini M, Carrillon Y (2021). Partial and complete repairs of massive rotator cuff tears maintain similar long-term improvements in clinical scores. Knee Surg Sport Traumatol Arthrosc.

[16] Cavalier M, Jullion S, Kany J, Grimberg J, Lefebvre Y, Oudet D (2018). Management of massive rotator cuff tears: prospective study in 218 patients. Orthop Traumatol Surg Res.

[17] Chen KH, Chiang ER, Wang HY, Ma HL (2017). Arthroscopic partial repair of irreparable rotator cuff tears: factors related to greater degree of clinical improvement at 2 years of follow-up. Arthrosc J Arthrosc Relat Surg.

[18] Cuff DJ, Pupello DR, Santoni BG (2016). Partial rotator cuff repair and biceps tenotomy for the treatment of patients with massive cuff tears and retained overhead elevation: midterm outcomes with a minimum 5 years of follow-up. J Shoulder Elb Surg.

[19] Di Benedetto ED, Di Benedetto P, Fiocchi A, Beltrame A, Causero A (2017). Partial repair in irreparable rotator cuff tear: our experience in long-term follow-up. Acta Biomed.

[20] Farazdaghi A, Paschos NK, Kelly JD (2021). Comparison between partial and full coverage repair in massive rotator cuff tears. A minimum five year follow-up. Orthop Traumatol Surg Res.

[21] Franceschi F, Papalia R, Vasta S, Leonardi F, Maffulli N, Denaro V (2015). Surgical management of irreparable rotator cuff tears. Knee Surg Sports Traumatol Arthrosc.

[22] Galasso O, Riccelli DA, De Gori M, De Benedetto M, Orlando N, Gasparini G (2017). Quality of life and functional results of arthroscopic partial repair of irreparable rotator cuff tears. Arthrosc J Arthrosc Relat Surg.

[23] Greiner S, Kaeaeb M, Voss A, Lawton R, Bhide P, Achenbach L (2021). Comparison of superior capsular reconstruction and partial infraspinatus repair: a matched-pair analysis of irreparable rotator cuff tears. Orthop J Sport Med.

[24] Heuberer PR, Kolblinger R, Buchleitner S, Pauzenberger L, Laky B, Auffarth A (2016). Arthroscopic management of massive rotator cuff tears: an evaluation of debridement, complete, and partial repair with and without force couple restoration. Knee Surg Sports Traumatol Arthrosc.

[25] Holtby R, Razmjou H (2014). Relationship between clinical and surgical findings and reparability of large and massive rotator cuff tears: a longitudinal study. BMC Musculoskelet Disord.

[26] Jeong J, Kim S, Yoon T, Eum K, Chun Y (2020). Arthroscopic repair of large and massive rotator cuff tears. J Bone Jt Surg Am.

[27] Kim S-J, Lee I-S, Kim S-H, Lee W-Y, Chun Y-M (2012). Arthroscopic partial repair of irreparable large to massive rotator cuff tears. Arthroscopy.

[28] Kim SJ, Kim SH, Lee SK, Seo JW, Chun YM (2013). Arthroscopic repair of massive contracted rotator cuff tears: aggressive release with anterior and posterior interval slides do not improve cuff healing and integrity. J Bone Jt Surg Ser A.

[29] Lee KW, Lee GS, Yang DS, Park SH, Chun YS, Choy WS (2020). Clinical outcome of arthroscopic partial repair of large to massive posterosuperior rotator cuff tears: medialization of the attachment site of the rotator cuff tendon. CiOS Clin Orthop Surg.

[30] Malahias MA, Brilakis E, Avramidis G, Trellopoulos A, Antonogiannakis E (2020). Arthroscopic partial repair with versus without biodegradable subacromial spacer for patients with massive rotator cuff tears: a case–control study. Musculoskelet Surg.

[31] Mori D, Funakoshi N, Yamashita F (2013). Arthroscopic surgery of irreparable large or massive rotator cuff tears with low-grade fatty degeneration of the infraspinatus: patch autograft procedure versus partial repair procedure. Arthrosc J Arthrosc Relat Surg.

[32] Moser M, Jablonski MV, Horodyski MB, Wright TW (2007). Functional outcome of surgically treated massive rotator cuff tears: a comparison of complete repair, partial repair, and debridement. Orthopedics.

[33] Pandey R, Tafazal S, Shyamsundar S, Modi A, Singh HP (2017). Outcome of partial repair of massive rotator cuff tears with and without human tissue allograft bridging repair. Shoulder Elb.

[34] Park SR, Sun DH, Kim J, Lee HJ, Bin KJ, Kim YS (2018). Is augmentation with the long head of the biceps tendon helpful in arthroscopic treatment of irreparable rotator cuff tears?. J Shoulder Elb Surg..

[35] Paribelli G, Boschi S, Randelli P, Compagnoni R, Leonardi F, Cassarino AM (2015). Clinical outcome of latissimus dorsi tendon transfer and partial cuff repair in irreparable postero-superior rotator cuff tear. Musculoskelet Surg.

[36] Porcellini G, Castagna A, Cesari E, Merolla G, Pellegrini A, Paladini P (2011). Partial repair of irreparable supraspinatus tendon tears: Clinical and radiographic evaluations at long-term follow-up. J Shoulder Elb Surg.

[37] Shon MS, Koh KH, Lim TK, Kim WJ, Kim KC, Yoo JC (2015). Arthroscopic partial repair of irreparable rotator cuff tears: preoperative factors associated with outcome deterioration over 2 years. Am J Sports Med.

[38] Wellmann M, Lichtenberg S, Da Silva G, Magosch P, Habermeyer P (2013). Results of arthroscopic partial repair of large retracted rotator cuff tears. Arthrosc J Arthrosc Relat Surg.

[39] Audenaert E, VanNuffel J, Schepens A, Verhelst M, Verdonk R (2006). Reconstruction of massive rotator cuff lesions with a synthetic interposition graft: a prospective study of 41 patients. Knee Surg Sport Traumatol Arthrosc.

[40] Badhe SP, Lawrence TM, Smith FD, Lunn PG (2008). An assessment of porcine dermal xenograft as an augmentation graft in the treatment of extensive rotator cuff tears. J Shoulder Elb Surg.

[41] Bond JL, Dopirak RM, Higgins J, Burns J, Snyder SJ (2008). Arthroscopic replacement of massive, irreparable rotator cuff tears using a graftjacket allograft: technique and preliminary results. Arthrosc J Arthrosc Relat Surg.

[42] Dukan R, Bommier A, Rousseau MA, Boyer P (2019). Superior capsule reconstruction for irreparable rotator cuff tear with a porcine dermal graft: preliminary results at 2 years minimum follow-up. Muscles Ligaments Tendons J.

[43] Gupta AK, Hug K, Berkoff DJ, Boggess BR, Gavigan M, Malley PC (2012). Dermal tissue allograft for the repair of massive irreparable rotator cuff tears. Am J Sports Med.

[44] Gupta AK, Hug K, Boggess B, Gavigan M, Toth AP (2013). Massive or 2-tendon rotator cuff tears in active patients with minimal glenohumeral arthritis: clinical and radiographic outcomes of reconstruction using dermal tissue matrix xenograft. Am J Sports Med.

[45] Kim JO, Lee JH, Kim KS, Ji JH, Koh SJ, Lee JH (2017). Rotator cuff bridging repair using acellular dermal matrix in large to massive rotator cuff tears: histologic and clinical analysis. J Shoulder Elb Surg.

[46] Kokkalis ZT, Mavrogenis AF, Scarlat M, Christodoulou M, Vottis C, Papagelopoulos PJ (2014). Human dermal allograft for massive rotator cuff tears. Orthopedics.

[47] Modi A, Singh HP, Pandey R, Armstrong A (2013). Management of irreparable rotator cuff tears with the graftjacket allograft as an interpositional graft. Shoulder Elb.

[48] Nada AM, Debnath UK, Robinson DA, Jordan C (2010). Treatment of massive rotator-cuff tears with a polyester ligament (Dacron) augmentation: clinical outcome. J Bone Jt Surg Ser B.

[49] Petrie MJ, Ismaiel AH (2013). Treatment of massive rotator-cuff tears with a polyester ligament (LARS) patch. Acta Orthop Belg.

[50] Rhee YG, Nam SC, Chan TL, Jin WY, Vishvanathan T (2008). Bridging the gap in immobile massive rotator cuff tears: augmentation using the tenotomized biceps. Am J Sports Med.

[51] Rhee SM, Oh JH (2017). Bridging graft in irreparable massive rotator cuff tears: autogenic biceps graft versus allogenic dermal patch graft. CiOS Clin Orthop Surg.

[52] Sano H, Mineta M, Kita A, Itoi E (2010). Tendon patch grafting using the long head of the biceps for irreparable massive rotator cuff tears. J Orthop Sci.

[53] Dimitrios V, Athanasios P, Eleni A, Xenofon P, George F, John F (2015). Results of reconstruction of massive irreparable rotator cuff tears using a fascia lata allograft. Indian J Orthop.

[54] Wong I, Burns J, Snyder S (2010). Arthroscopic GraftJacket repair of rotator cuff tears. J Shoulder Elb Surg.

[55] Alarcon J, Uribe-Echevarria B, Clares C, Apablaza D, Vargas J, Benavente S, et al. Superior capsular reconstruction with autologous fascia lata using single lateral row technique is an effective option in massive irreparable rotator cuff tears. Minimum two-year follow-up. Sci Total Environ. 2021;142972. 10.1016/j.arthro.2021.04.009 33957215

[56] Barth J, Olmos MI, Swan J, Barthelemy R, Delsol P, Boutsiadis A (2020). Superior capsular reconstruction with the long head of the biceps autograft prevents infraspinatus retear in massive posterosuperior retracted rotator cuff tears. Am J Sports Med.

[57] Burkhart SS, Pranckun JJ, Hartzler RU (2020). Superior capsular reconstruction for the operatively irreparable rotator cuff tear: clinical outcomes are maintained 2 years after surgery. Arthrosc - J Arthrosc Relat Surg.

[58] Denard PJ, Brady PC, Adams CR, Tokish JM, Burkhart SS (2018). Preliminary results of arthroscopic superior capsule reconstruction with dermal allograft. Arthrosc J Arthrosc Relat Surg.

[59] Ferrando A, Kingston R, Delaney RA (2021). Superior capsular reconstruction using a porcine dermal xenograft for irreparable rotator cuff tears: outcomes at minimum two-year follow-up. J Shoulder Elb Surg.

[60] Kim D, Jaewoong U, Lee J, Kim J (2021). Improved clinical and radiographic outcomes seen after superior capsule reconstruction using long head biceps tendon allograft. Arthrosc J Arthrosc Relat Surg.

[61] Kocaoglu B, Firatli G, Ulku TK (2020). Partial rotator cuff repair with superior capsular reconstruction using the biceps tendon is as effective as superior capsular reconstruction using a tensor fasciae latae autograft in the treatment of irreparable massive rotator cuff tears. Orthop J Sport Med.

[62] LaBelle MW, Mengers S, Strony J, Peck M, Flannery R, Cupp S (2021). Evaluating the role of graft integrity on outcomes: clinical and imaging results following superior capsular reconstruction. J Shoulder Elbow Surg.

[63] Lacheta L, Horan MP, Schairer WW, Goldenberg BT, Dornan GJ, Pogorzelski J (2020). Clinical and imaging outcomes after arthroscopic superior capsule reconstruction with human dermal allograft for irreparable posterosuperior rotator cuff tears: a minimum 2-year follow-up. Arthrosc - J Arthrosc Relat Surg.

[64] Lee SJ, Min YK (2018). Can inadequate acromiohumeral distance improvement and poor posterior remnant tissue be the predictive factors of re-tear? Preliminary outcomes of arthroscopic superior capsular reconstruction. Knee Surg Sports Traumatol Arthrosc.

[65] Lim S, AlRamadhan H, Kwak JM, Hong H, Jeon IH (2019). Graft tears after arthroscopic superior capsule reconstruction (ASCR): pattern of failure and its correlation with clinical outcome. Arch Orthop Trauma Surg.

[66] Okamura K, Abe M, Yamada Y, Makihara T, Yoshimizu T, Sakaki Y (2021). Arthroscopic superior capsule reconstruction with Teflon felt synthetic graft for irreparable massive rotator cuff tears: clinical and radiographic results at minimum 2-year follow-up. J Shoulder Elb Surg [Internet].

[67] Ohta S, Komai O, Onochi Y (2020). Outcomes of superior capsule reconstruction for massive rotator cuff tears and risk factors for postoperative retear. Arch Orthop Trauma Surg.

[68] Ozturk BY, Ak S, Gultekin O, Baykus A, Kulduk A (2021). Prospective, randomized evaluation of latissimus dorsi transfer and superior capsular reconstruction in massive, irreparable rotator cuff tears. J Shoulder Elb Surg.

[69] Pashuck TD, Hirahara AM, Cook JL, Cook CR, Andersen WJ, Smith MJ (2021). Superior capsular reconstruction using dermal allograft is a safe and effective treatment for massive irreparable rotator cuff tears: 2-year clinical outcomes. Arthrosc J Arthrosc Relat Surg.

[70] Pennington WT, Bartz BA, Pauli JM, Walker CE, Schmidt W (2018). Arthroscopic superior capsular reconstruction with acellular dermal allograft for the treatment of massive irreparable rotator cuff tears: short-term clinical outcomes and the radiographic parameter of superior capsular distance. Arthroscopy.

[71] Polacek M (2019). Arthroscopic superior capsular reconstruction with acellular porcine dermal xenograft for the treatment of massive irreparable rotator cuff tears. Arthrosc Sport Med Rehabil.

[72] Polacek M, Nyegaard CP (2020). Superior capsular reconstruction using 3-layered fascia lata autograft reinforced with a nonresorbable suture mesh. Arthrosc Sport Med Rehabil.

[73] Takayama K, Yamada S, Kobori Y (2021). Clinical effectiveness of mini-open superior capsular reconstruction using autologous tensor fascia lata graft. J Shoulder Elb Surg.

[74] Blanke F, Sachser J, Majewski M (2017). Operative techniques for treatment of chronic massive rotator cuff lesions: deltoid flap transfer versus arthroscopic debridement. Acta Orthop Belg.

[75] Boileau P, Baque F, Valerio L, Ahrens P, Chuinard C, Trojani C (2007). Isolated arthroscopic biceps tenotomy or tenodesis improves symptoms in patients with massive irreparable rotator cuff tears. J Bone Joint Surg Am.

[76] Klinger HM, Steckel H, Ernstberger T, Baums MH (2005). Arthroscopic debridement of massive rotator cuff tears: negative prognostic factors. Arch Orthop Trauma Surg.

[77] Lee BG, Cho NS, Rhee YG (2011). Results of arthroscopic decompression and tuberoplasty for irreparable massive rotator cuff tears. Arthrosc J Arthrosc Relat Surg.

[78] Mirzaee F, Aslani M, Zafarani Z, Aslani H (2019). Treatment of massive irreparable rotator cuff tear. Arch Bone Jt Surg.

[79] Park JG, Cho NS, Song JH, Baek JH, Rhee YG (2016). Long-term outcome of tuberoplasty for irreparable massive rotator cuff tears: Is tuberoplasty really applicable?. J shoulder Elb Surg.

[80] Scheibel M, Lichtenberg S, Habermeyer P (2004). Reversed arthroscopic subacromial decompression for massive rotator cuff tears. J Shoulder Elb Surg.

[81] Vad VB, Warren RF, Altchek DW, O’Brien SJ, Rose HA, Wickiewicz TL (2002). Negative prognostic factors in managing massive rotator cuff tears. Clin J Sport Med.

[82] de Castro Veado MA, Rodrigues AU (2010). Functional evaluation of patients who have undergone arthroscopic debridement to treat massive and irreparable tears of the rotator cuff. Rev Bras Ortop (English Ed.).

[83] Verhelst L, Vandekerckhove PJ, Sergeant G, Liekens K, Van Hoonacker P, Berghs B (2010). Reversed arthroscopic subacromial decompression for symptomatic irreparable rotator cuff tears: mid-term follow-up results in 34 shoulders. J Shoulder Elb Surg.

[84] Basat H, Kirçil C, Armangil M, Demirtş M (2017). Treatment alternative for irreparable rotator cuff ruptures: arthroscopic biodegradable balloon. Niger J Clin Pract.

[85] Bakti N, Bhat M, Gulihar A, Prasad V, Singh B, Princess O (2019). Subacromial balloon interpositional arthroplasty for the management of irreparable rotator cuff tears : five-year results. Open Orthop J.

[86] Deranlot J, Herisson O, Nourissat G, Zibili D, Werthel JD, Vigan M (2017). Arthroscopic subacromial spacer implantation in patients with massive irreparable rotator cuff tears: clinical and radiographic results of 39 retrospective cases. Arthrosc J Arthrosc Relat Surg.

[87] Familiari F, Nayar SK, Russo R, De Gori M, Ranuccio F, Mastroianni V (2021). Subacromial balloon spacer for massive, irreparable rotator cuff tears is associated with improved shoulder function and high patient satisfaction. Arthrosc J Arthrosc Relat Surg.

[88] Gervasi E, Maman E, Dekel A, Cautero E (2016). Fluoroscopy-guided biodegradable spacer implantation using local anesthesia: safety and efficacy study in patients with massive rotator cuff tears. Musculoskelet Surg.

[89] Gervasi E, Maman E, Dekel A, Markovitz E, Cautero E (2021). Fluoroscopically guided subacromial spacer implantation for massive rotator cuff tears: two years of prospective follow-up. Orthop J Sport Med.

[90] Ruiz Ibán MA, Lorente Moreno R, Ruiz Díaz R, Álvarez Sciamanna R, Paniagua Gonzalez A, Lorente Gómez A (2018). The absorbable subacromial spacer for irreparable posterosuperior cuff tears has inconsistent results. Knee Surg Sport Traumatol Arthrosc.

[91] Lorente Gómez A, Ruiz Ibán MÁ, Ruiz Díaz R, Vega Rodríguez RM, Álvarez R, Paniagua A (2017). Malos resultados a corto plazo del balón subacromial InSpace®. Resultados de 15 casos consecutivos con un año de seguimiento. Rev Española Artrosc y Cirugía Articul..

[92] Maman E, Safran O, Beyth S, Mozes G, Dekel A, Michael B (2017). Biceps tenotomy does not affect the functional outcomes of patients treated with spacer implantation due to massive irreparable rotator cuff tears. Open Orthop J.

[93] Senekovic V, Poberaj B, Kovacic L, Mikek M, Adar E, Dekel A (2013). Prospective clinical study of a novel biodegradable sub-acromial spacer in treatment of massive irreparable rotator cuff tears. Eur J Orthop Surg Traumatol.

[94] Metcalfe A, Parsons H, Parsons N, Brown J, Fox J, Gemperlé Mannion E (2022). Subacromial balloon spacer for irreparable rotator cuff tears of the shoulder (START:REACTS): a group-sequential, double-blind, multicentre randomised controlled trial. Lancet.

[95] Verma NN, Srikumaran U, Roden C, Rogusky E, Lapner P, Heather N, Abboud J (2022). Inspace implant compared with partial repair for the treatment of full-thickness massive rotator cuff tears. J Bone Jt Surg.

[96] Sevivas N, Ferreira N, Andrade R, Moreira P, Alves D (2017). Reverse shoulder arthroplasty for irreparable massive rotator cuff tears : a systematic review with meta-analysis and meta-regression. J Shoulder Elb Surg.

[97] Ernstbrunner L, Andronic O, Grubhofer F, Camenzind RS, Wieser K, Gerber C (2019). Long-term results of reverse total shoulder arthroplasty for rotator cuff dysfunction: A systematic review of longitudinal outcomes. J Shoulder Elb Surg.

[98] Kääb MJ, Kohut G, Irlenbusch U, Joudet T, Reuther F (2021). Reverse total shoulder arthroplasty in massive rotator cuff tears: Does the Hamada classification predict clinical outcomes?. Arch Orthop Trauma Surg.

[99] Viswanath A, Bale S, Trail I (2021). Reverse total shoulder arthroplasty for irreparable rotator cuff tears without arthritis: a systematic review. J Clin Orthop Trauma.

[100] Reed M, Brittain R, Howard P, Laurence S, Stonadge J, Wilkinson M et al. 16th Annual Report. The National Joint Registry 18th Annual Report 2021. London: National Joint Registry; 2021 Sep.

[101] Adam JR, Nanjayan SKT, Johnson M, Rangan A (2021). Tendon transfers for irreparable rotator cuff tears. J Clin Orthop Trauma.

[102] Clark NJ, Elhassan BT (2018). The role of tendon transfers for irreparable rotator cuff tears. Curr Rev Musculoskelet Med.

[103] Gerber C, Rahm SA, Catanzaro S, Farshad M, Moor BK (2013). Latissimus dorsi tendon transfer for treatment of irreparable posterosuperior rotator cuff tears: long-term results at a minimum follow-up of ten years. J Bone Jt Surg Ser A.

[104] Kovacevic D, Suriani RJ, Grawe BM, Yian EH, Gilotra MN, Hasan SA (2020). Management of irreparable massive rotator cuff tears: a systematic review and meta-analysis of patient-reported outcomes, reoperation rates, and treatment response. J Shoulder Elb Surg.

[105] Kooistra B, Gurnani N, Weening A, van den Bekerom M, van Deurzen D (2019). Low level of evidence for all treatment modalities for irreparable posterosuperior rotator cuff tears. Knee Surg Sport Traumatol Arthrosc.

[106] Levy O, Mullett H, Roberts S, Copeland S (2008). The role of anterior deltoid reeducation in patients with massive irreparable degenerative rotator cuff tears. J Shoulder Elb Surg.

